# Supplementary material for: A Multicentre Evaluation of Dosiomics Features Reproducibility, Stability and Sensitivity
Source: Cancers (Basel). 2021 Jul 30;13(15):3835. doi: 10.3390/cancers13153835 (PMC8345157; doi:10.3390/cancers13153835)
Supplement: Supplementary file 1 [file cancers-13-03835-s001.zip › FigsSM.pdf]

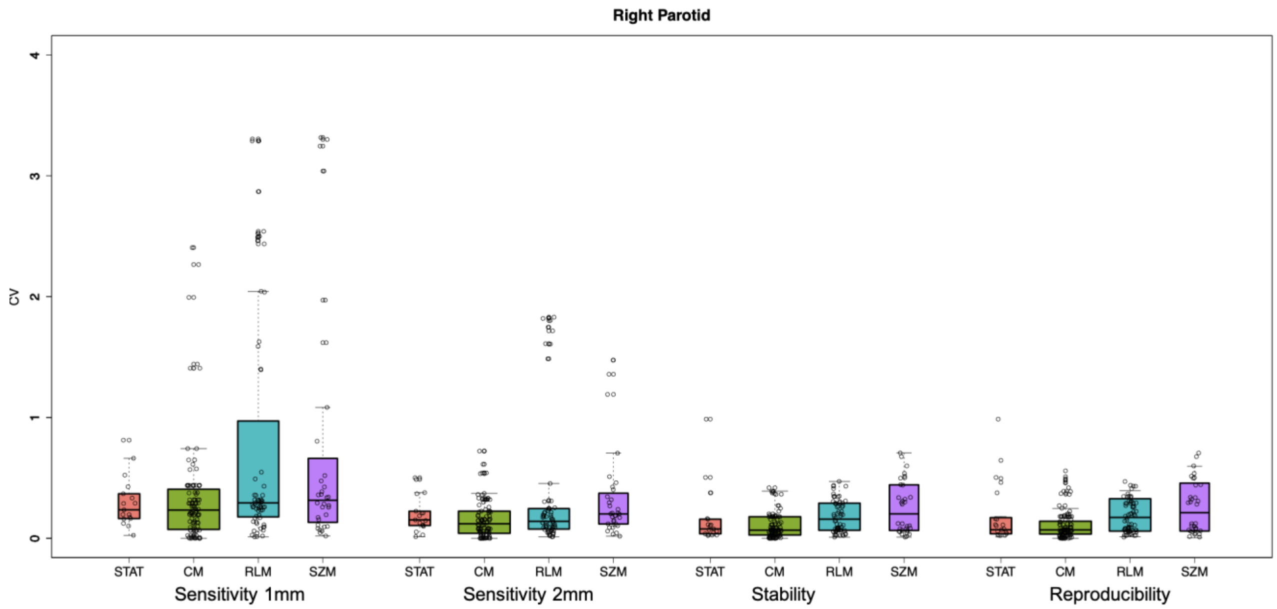

Figure. S1 – Box plot of the CV values for the sensitivity (1 mm and 2 mm), stability and reproducibility studies, grouped for the four different features' families (STAT, CM, RLM and GSZ) for the ROI right parotid.

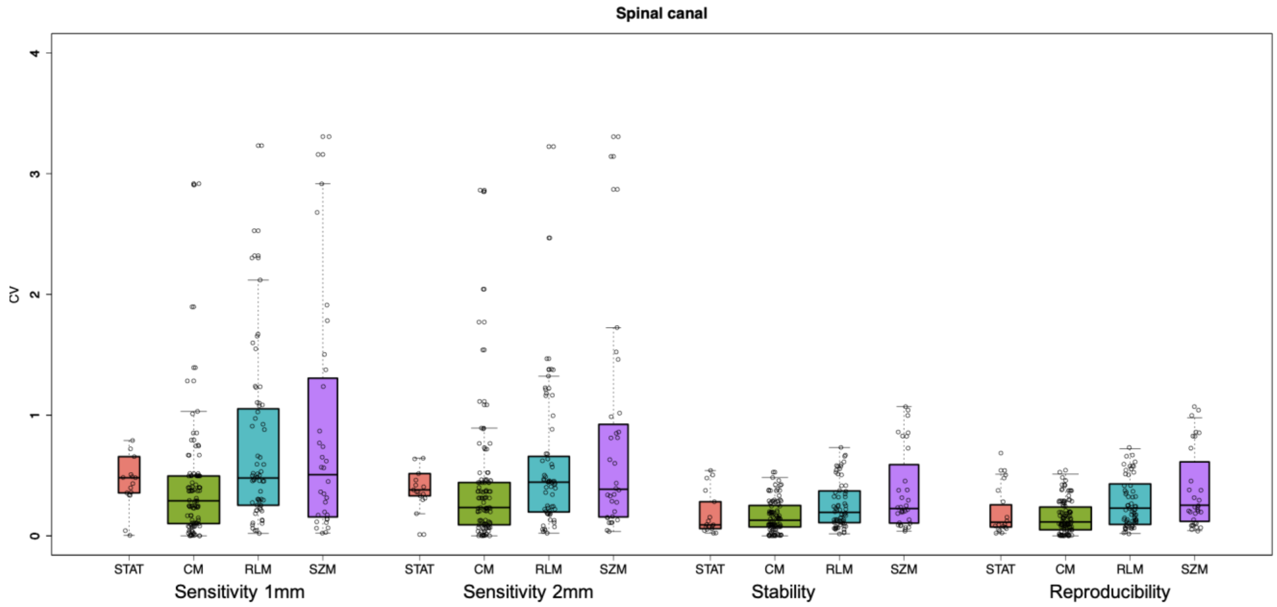

FigureS2 – Box plot of the CV values for the sensitivity (1 mm and 2 mm), stability and reproducibility studies, grouped for the four different features' families (STAT, CM, RLM and GSZ) for the ROI spinal canal.

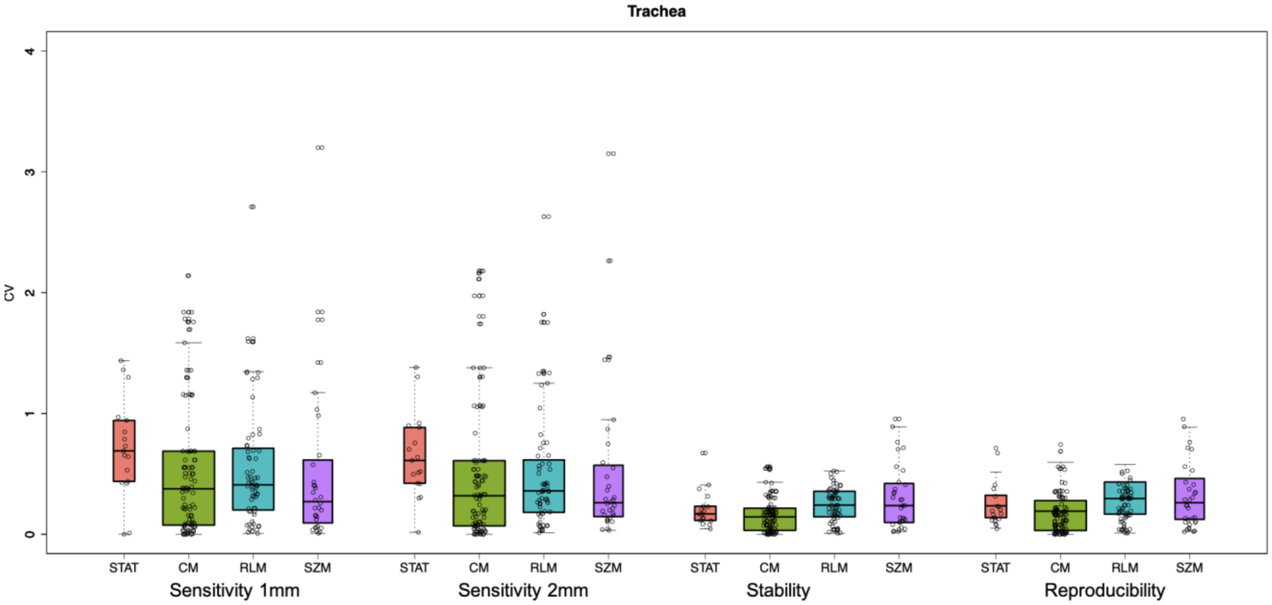

Figure S3 – Box plot of the CV values for the sensitivity (1 mm and 2 mm), stability and reproducibility studies, grouped for the four different features' families (STAT, CM, RLM and GSZ) for the ROI trachea.

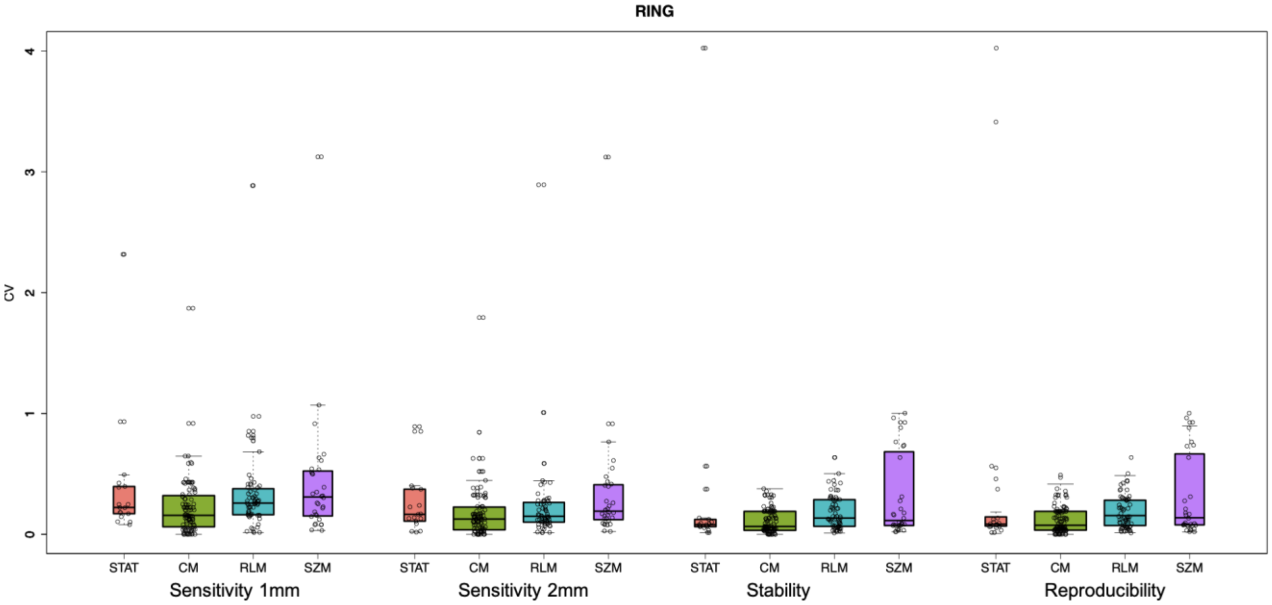

Figure S4 – Box plot of the CV values for the sensitivity (1 mm and 2 mm), stability and reproducibility studies, grouped for the four different features' families (STAT, CM, RLM and GSZ) for the ROI RING.

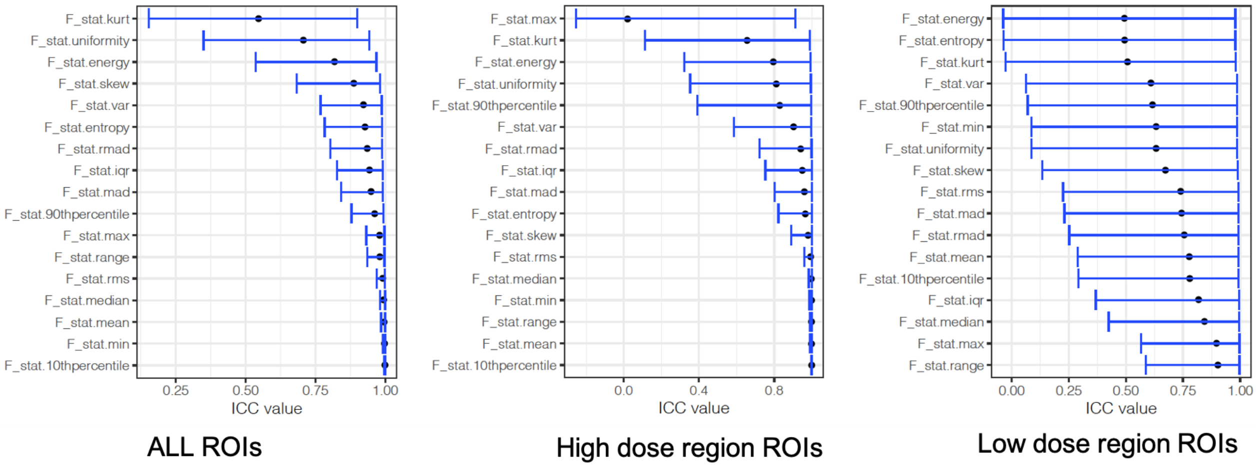

Figure S5 – Example of ICC results for the STAT dosiomic features' family and considering the three possible ROIs groups: all ROIs, high dose region ROIs and low dose region ROIs.
