# Supplementary material for: A Multicentre Evaluation of Dosiomics Features Reproducibility, Stability and Sensitivity
Source: Cancers (Basel). 2021 Jul 30;13(15):3835. doi: 10.3390/cancers13153835 (PMC8345157; doi:10.3390/cancers13153835)
Supplement: Supplementary file 1 [file cancers-13-03835-s001.zip › Table S1.pdf]

Table S1. Reproducibility CV values for all the dosiomic features employed in the study and for all the six ROIs: left parotid, right parotid, PTV, RING, Spinal Canal and Trachea.

Reproducibility	Left Parotid	Right Parotid	PTV	RING	Spinal Canal	Trachea
F_stat.mean	0.15	0.04	0.01	0.07	0.11	0.22
F_stat.var	0.45	0.07	0.60	0.14	0.11	0.44
F_stat.skew	3.13	0.05	0.30	0.08	0.51	0.17
F_stat.kurt	4.11	0.64	0.65	3.41	0.69	0.51
F_stat.median	0.25	0.09	0.01	0.18	0.17	0.17
F_stat.min	0.30	0.17	0.02	0.08	0.15	0.08
F_stat.10thpercentile	0.33	0.18	0.02	0.05	0.26	0.09
F_stat.90thpercentile	0.07	0.01	0.01	0.09	0.07	0.28
F_stat.max	0.08	0.01	0.01	0.01	0.02	0.14
F_stat.iqr	0.33	0.09	0.41	0.11	0.09	0.32
F_stat.range	0.09	0.01	0.09	0.01	0.02	0.14
F_stat.mad	0.28	0.05	0.35	0.08	0.07	0.27
F_stat.rmad	0.33	0.08	0.41	0.10	0.09	0.32
F_stat.energy	0.59	0.50	0.46	0.55	0.54	0.71
F_stat.rms	0.12	0.02	0.01	0.07	0.09	0.24
F_stat.entropy	0.05	0.05	0.05	0.04	0.05	0.05
F_stat.uniformity	0.46	0.46	0.46	0.46	0.46	0.46
F_cm.joint.max	0.13	0.07	0.10	0.07	0.13	0.08
F_cm.joint.avg	0.16	0.04	0.01	0.07	0.12	0.23
F_cm.joint.var	0.41	0.14	0.23	0.19	0.09	0.48
F_cm.joint.entr	0.05	0.02	0.11	0.03	0.06	0.16
F_cm.diff.avg	0.34	0.22	0.30	0.22	0.29	0.26
F_cm.diff.var	0.34	0.49	0.26	0.39	0.30	0.23
F_cm.diff.entr	0.20	0.11	0.18	0.12	0.16	0.16
F_cm.sum.avg	0.16	0.04	0.01	0.07	0.12	0.23
F_cm.sum.var	0.42	0.14	0.23	0.19	0.10	0.49
F_cm.sum.entr	0.03	0.03	0.09	0.05	0.03	0.16
F_cm.energy	0.09	0.11	0.12	0.08	0.27	0.10
F_cm.contrast	0.43	0.46	0.34	0.41	0.40	0.31
F_cm.dissimilarity	0.34	0.22	0.30	0.22	0.29	0.26
F_cm.inv.diff	0.04	0.06	0.03	0.03	0.07	0.02
F_cm.inv.diff.norm	0.01	0.00	0.00	0.00	0.00	0.00
F_cm.inv.diff.mom	0.04	0.07	0.03	0.03	0.08	0.02
F_cm.inv.diff.mom.norm	0.00	0.00	0.00	0.00	0.00	0.00
F_cm.inv.var	0.25	0.05	0.27	0.06	0.18	0.22
F_cm.corr	0.01	0.01	0.07	0.01	0.01	0.04
F_cm.auto.corr	0.26	0.05	0.02	0.14	0.18	0.42
F_cm.clust.tend	0.42	0.14	0.23	0.19	0.10	0.49

**F_cm.clust.shade**	2.04	0.15	0.44	0.23	0.40	0.53
**F_cm.clust.prom**	0.70	0.21	0.43	0.22	0.26	0.69
**F_cm.info.corr.1**	0.09	0.05	0.18	0.06	0.09	0.09
**F_cm.info.corr.2**	0.10	0.00	0.05	0.04	0.01	0.12
**F_cm_merged.joint.max**	0.13	0.07	0.10	0.07	0.13	0.08
**F_cm_merged.joint.avg**	0.16	0.04	0.01	0.07	0.12	0.23
**F_cm_merged.joint.var**	0.41	0.14	0.23	0.19	0.09	0.48
**F_cm_merged.joint.entr**	0.06	0.02	0.12	0.03	0.06	0.16
**F_cm_merged.diff.avg**	0.34	0.22	0.30	0.22	0.29	0.26
**F_cm_merged.diff.var**	0.36	0.48	0.27	0.39	0.31	0.24
**F_cm_merged.diff.entr**	0.21	0.10	0.18	0.12	0.17	0.16
**F_cm_merged.sum.avg**	0.16	0.04	0.01	0.07	0.12	0.23
**F_cm_merged.sum.var**	0.42	0.14	0.23	0.19	0.10	0.49
**F_cm_merged.sum.entr**	0.03	0.03	0.09	0.05	0.03	0.16
**F_cm_merged.energy**	0.09	0.11	0.12	0.08	0.28	0.10
**F_cm_merged.contrast**	0.43	0.46	0.34	0.41	0.40	0.31
**F_cm_merged.dissimilarity**	0.34	0.22	0.30	0.22	0.29	0.26
**F_cm_merged.inv.diff**	0.04	0.06	0.03	0.03	0.07	0.02
**F_cm_merged.inv.diff.norm**	0.01	0.00	0.00	0.00	0.00	0.00
**F_cm_merged.inv.diff.mom**	0.04	0.07	0.03	0.03	0.08	0.02
**F_cm_merged.inv.diff.mom.norm**	0.00	0.00	0.00	0.00	0.00	0.00
**F_cm_merged.inv.var**	0.25	0.05	0.27	0.06	0.18	0.22
**F_cm_merged.corr**	0.01	0.01	0.07	0.01	0.01	0.03
**F_cm_merged.auto.corr**	0.26	0.05	0.02	0.14	0.18	0.42
**F_cm_merged.clust.tend**	0.42	0.14	0.23	0.19	0.10	0.49
**F_cm_merged.clust.shade**	2.04	0.15	0.44	0.23	0.40	0.53
**F_cm_merged.clust.prom**	0.70	0.21	0.43	0.22	0.26	0.69
**F_cm_merged.info.corr.1**	0.11	0.08	0.19	0.07	0.11	0.10
**F_cm_merged.info.corr.2**	0.12	0.00	0.07	0.04	0.01	0.13
**F_cm_2.5D.joint.max**	0.46	0.25	0.37	0.27	0.21	0.26
**F_cm_2.5D.joint.avg**	0.12	0.04	0.03	0.02	0.06	0.02
**F_cm_2.5D.joint.var**	0.18	0.05	0.12	0.05	0.04	0.03
**F_cm_2.5D.joint.entr**	0.03	0.02	0.03	0.04	0.04	0.03
**F_cm_2.5D.diff.avg**	0.24	0.24	0.26	0.27	0.28	0.26
**F_cm_2.5D.diff.var**	0.69	0.56	0.45	0.47	0.54	0.43
**F_cm_2.5D.diff.entr**	0.12	0.11	0.10	0.14	0.13	0.10
**F_cm_2.5D.sum.avg**	0.12	0.04	0.03	0.02	0.06	0.02
**F_cm_2.5D.sum.var**	0.18	0.05	0.14	0.05	0.04	0.03
**F_cm_2.5D.sum.entr**	0.01	0.01	0.01	0.00	0.01	0.00
**F_cm_2.5D.energy**	0.39	0.13	0.30	0.26	0.30	0.20
**F_cm_2.5D.contrast**	0.57	0.51	0.47	0.49	0.51	0.47
**F_cm_2.5D.dissimilarity**	0.24	0.24	0.26	0.27	0.28	0.26

**F_cm_2.5D.inv.diff**	0.09	0.09	0.17	0.12	0.15	0.16
**F_cm_2.5D.inv.diff.norm**	0.04	0.01	0.01	0.01	0.02	0.03
**F_cm_2.5D.inv.diff.mom**	0.13	0.11	0.25	0.16	0.22	0.24
**F_cm_2.5D.inv.diff.mom.norm**	0.04	0.00	0.00	0.00	0.00	0.01
**F_cm_2.5D.inv.var**	0.12	0.08	0.20	0.10	0.18	0.25
**F_cm_2.5D.corr**	0.01	0.01	0.04	0.00	0.01	0.01
**F_cm_2.5D.auto.corr**	0.12	0.05	0.06	0.04	0.09	0.03
**F_cm_2.5D.clust.tend**	0.18	0.05	0.14	0.05	0.04	0.03
**F_cm_2.5D.clust.shade**	0.77	0.05	0.17	0.23	0.28	0.37
**F_cm_2.5D.clust.prom**	0.32	0.04	0.18	0.05	0.11	0.04
**F_cm_2.5D.info.corr.1**	0.08	0.09	0.18	0.10	0.13	0.10
**F_cm_2.5D.info.corr.2**	0.00	0.00	0.00	0.00	0.00	0.00
**F_cm.2.5Dmerged.joint.max**	0.16	0.24	0.10	0.17	0.21	0.23
**F_cm.2.5Dmerged.joint.avg**	0.15	0.04	0.01	0.07	0.12	0.23
**F_cm.2.5Dmerged.joint.var**	0.44	0.07	0.61	0.15	0.12	0.44
**F_cm.2.5Dmerged.joint.entr**	0.12	0.02	0.17	0.03	0.04	0.11
**F_cm.2.5Dmerged.diff.avg**	0.34	0.22	0.30	0.23	0.29	0.26
**F_cm.2.5Dmerged.diff.var**	0.39	0.48	0.27	0.41	0.30	0.28
**F_cm.2.5Dmerged.diff.entr**	0.24	0.11	0.18	0.13	0.16	0.18
**F_cm.2.5Dmerged.sum.avg**	0.15	0.04	0.01	0.07	0.12	0.23
**F_cm.2.5Dmerged.sum.var**	0.44	0.07	0.63	0.15	0.12	0.44
**F_cm.2.5Dmerged.sum.entr**	0.10	0.01	0.15	0.03	0.01	0.11
**F_cm.2.5Dmerged.energy**	0.27	0.13	0.24	0.24	0.19	0.27
**F_cm.2.5Dmerged.contrast**	0.43	0.46	0.33	0.42	0.40	0.31
**F_cm.2.5Dmerged.dissimilarity**	0.34	0.22	0.30	0.23	0.29	0.26
**F_cm.2.5Dmerged.inv.diff**	0.04	0.06	0.03	0.04	0.07	0.02
**F_cm.2.5Dmerged.inv.diff.norm**	0.01	0.00	0.00	0.00	0.00	0.00
**F_cm.2.5Dmerged.inv.diff.mom**	0.04	0.08	0.03	0.04	0.08	0.02
**F_cm.2.5Dmerged.inv.diff.mom.norm**	0.00	0.00	0.00	0.00	0.00	0.00
**F_cm.2.5Dmerged.inv.var**	0.25	0.05	0.27	0.06	0.18	0.22
**F_cm.2.5Dmerged.corr**	0.01	0.00	0.04	0.00	0.00	0.00
**F_cm.2.5Dmerged.auto.corr**	0.24	0.05	0.02	0.14	0.19	0.42
**F_cm.2.5Dmerged.clust.tend**	0.44	0.07	0.63	0.15	0.12	0.44
**F_cm.2.5Dmerged.clust.shade**	1.60	0.08	0.72	0.17	0.34	0.60
**F_cm.2.5Dmerged.clust.prom**	0.71	0.09	0.66	0.19	0.16	0.74
**F_cm.2.5Dmerged.info.corr.1**	0.08	0.09	0.10	0.05	0.09	0.03
**F_cm.2.5Dmerged.info.corr.2**	0.12	0.00	0.04	0.00	0.00	0.02
**F_rlm.sre**	0.02	0.02	0.02	0.05	0.03	0.01
**F_rlm.lre**	0.17	0.18	0.21	0.30	0.27	0.12
**F_rlm.lgre**	0.41	0.19	0.32	0.10	0.23	0.19
**F_rlm.hgre**	0.08	0.08	0.05	0.03	0.09	0.03
**F_rlm.srlge**	0.38	0.35	0.32	0.17	0.26	0.20

**F_rlm.srhge**	0.09	0.08	0.03	0.06	0.08	0.03
**F_rlm.lrlge**	0.60	0.33	0.30	0.49	0.50	0.14
**F_rlm.lrhge**	0.09	0.21	0.31	0.26	0.25	0.16
**F_rlm.glnu**	0.30	0.31	0.42	0.39	0.37	0.44
**F_rlm.glnu.norm**	0.23	0.14	0.01	0.03	0.06	0.02
**F_rlm.rlnu**	0.47	0.39	0.39	0.31	0.37	0.42
**F_rlm.rlnu.norm**	0.04	0.05	0.04	0.10	0.07	0.02
**F_rlm.r.perc**	0.08	0.08	0.16	0.17	0.08	0.04
**F_rlm.gl.var**	0.15	0.06	0.02	0.05	0.05	0.06
**F_rlm.rl.var**	0.34	0.33	0.72	0.48	0.56	0.48
**F_rlm.rl.entr**	0.04	0.04	0.02	0.03	0.03	0.01
**F_rlm_merged.sre**	0.42	0.03	0.18	0.07	0.16	0.24
**F_rlm_merged.lre**	0.38	0.17	0.48	0.36	0.58	0.36
**F_rlm_merged.lgre**	0.33	0.38	0.02	0.09	0.41	0.18
**F_rlm_merged.hgre**	0.19	0.07	0.02	0.13	0.18	0.43
**F_rlm_merged.srlge**	0.55	0.37	0.18	0.21	0.39	0.27
**F_rlm_merged.srhge**	0.38	0.07	0.18	0.07	0.13	0.52
**F_rlm_merged.lrlge**	0.44	0.33	0.48	0.35	0.72	0.28
**F_rlm_merged.lrhge**	0.69	0.23	0.48	0.44	0.54	0.57
**F_rlm_merged.glnu**	0.31	0.28	0.24	0.27	0.24	0.24
**F_rlm_merged.glnu.norm**	0.08	0.17	0.09	0.09	0.16	0.12
**F_rlm_merged.rlnu**	0.28	0.36	0.18	0.23	0.09	0.41
**F_rlm_merged.rlnu.norm**	0.34	0.06	0.26	0.13	0.26	0.22
**F_rlm_merged.r.perc**	0.36	0.11	0.38	0.22	0.25	0.18
**F_rlm_merged.gl.var**	0.38	0.13	0.14	0.11	0.12	0.44
**F_rlm_merged.rl.var**	0.34	0.24	0.52	0.41	0.59	0.47
**F_rlm_merged.rl.entr**	0.06	0.04	0.06	0.05	0.06	0.08
**F_rlm_2.5D.sre**	0.44	0.03	0.17	0.08	0.16	0.24
**F_rlm_2.5D.lre**	0.45	0.20	0.50	0.28	0.56	0.37
**F_rlm_2.5D.lgre**	0.35	0.37	0.02	0.19	0.24	0.32
**F_rlm_2.5D.hgre**	0.17	0.06	0.02	0.11	0.19	0.44
**F_rlm_2.5D.srlge**	0.58	0.37	0.17	0.28	0.46	0.43
**F_rlm_2.5D.srhge**	0.45	0.05	0.17	0.05	0.11	0.50
**F_rlm_2.5D.lrhge**	0.72	0.26	0.51	0.45	0.61	0.58
**F_rlm_2.5D.glnu**	0.37	0.37	0.32	0.35	0.23	0.18
**F_rlm_2.5D.glnu.norm**	0.19	0.05	0.20	0.05	0.09	0.39
**F_rlm_2.5D.rlnu**	0.28	0.36	0.20	0.24	0.08	0.45
**F_rlm_2.5D.rlnu.norm**	0.34	0.07	0.28	0.14	0.27	0.28
**F_rlm_2.5D.gl.var**	0.39	0.08	0.31	0.09	0.11	0.40
**F_rlm_2.5D.rl.var**	0.37	0.27	0.54	0.30	0.60	0.39
**F_rlm_2.5D.rl.entr**	0.03	0.02	0.05	0.03	0.07	0.06
**F_rlm_2.5D.lrlrlm_25D_merged.dfge**	0.44	0.33	0.50	0.25	0.63	0.32

**F_rlm.2.5Dmerged.sre**	0.43	0.03	0.17	0.07	0.15	0.23
**F_rlm.2.5Dmerged.lre**	0.46	0.18	0.50	0.28	0.50	0.36
**F_rlm.2.5Dmerged.lgre**	0.34	0.37	0.02	0.19	0.24	0.33
**F_rlm.2.5Dmerged.hgre**	0.17	0.06	0.02	0.11	0.18	0.44
**F_rlm.2.5Dmerged.srlge**	0.58	0.36	0.17	0.27	0.45	0.43
**F_rlm.2.5Dmerged.srhge**	0.43	0.05	0.17	0.05	0.11	0.51
**F_rlm.2.5Dmerged.lrlge**	0.41	0.34	0.50	0.24	0.63	0.31
**F_rlm.2.5Dmerged.lrhge**	0.74	0.23	0.50	0.44	0.55	0.56
**F_rlm.2.5Dmerged.glnu**	0.38	0.37	0.32	0.35	0.23	0.18
**F_rlm.2.5Dmerged.glnu.norm**	0.19	0.05	0.21	0.06	0.09	0.39
**F_rlm.2.5Dmerged.rlnu**	0.27	0.36	0.20	0.24	0.08	0.45
**F_rlm.2.5Dmerged.rlnu.norm**	0.35	0.06	0.28	0.14	0.26	0.28
**F_rlm.2.5Dmerged.r.perc**	0.27	0.06	0.26	0.11	0.22	0.20
**F_rlm.2.5Dmerged.gl.var**	0.40	0.09	0.32	0.10	0.11	0.40
**F_rlm.2.5Dmerged.rl.var**	0.38	0.25	0.53	0.29	0.57	0.38
**F_rlm.2.5Dmerged.rl.entr**	0.03	0.02	0.05	0.03	0.07	0.05
**F_szm.sze**	1.30	0.05	0.15	0.09	0.23	0.33
**F_szm.lze**	0.87	0.49	0.85	0.67	0.82	0.71
**F_szm.lgze**	0.28	0.39	0.02	0.08	0.38	0.18
**F_szm.hgze**	0.17	0.07	0.02	0.09	0.13	0.41
**F_szm.szlge**	0.73	0.40	0.14	0.72	0.38	0.43
**F_szm.szhge**	1.41	0.07	0.16	0.07	0.30	0.62
**F_szm.lzlge**	1.04	0.31	0.85	0.66	0.72	0.67
**F_szm.lzhge**	1.15	0.59	0.85	0.71	0.98	0.85
**F_szm.glnu**	0.37	0.30	0.08	0.16	0.10	0.25
**F_szm.glnu.norm**	0.12	0.10	0.11	0.10	0.17	0.13
**F_szm.zsnu**	0.87	0.28	0.22	0.22	0.33	0.43
**F_szm.zsnu.norm**	0.44	0.10	0.16	0.11	0.18	0.14
**F_zsm.z.perc**	0.72	0.14	0.55	0.39	0.51	0.47
**F_szm.gl.var**	0.20	0.11	0.10	0.07	0.09	0.45
**F_szm.zs.var**	0.79	0.52	0.84	0.82	0.98	0.89
**F_szm.z.entr**	0.07	0.04	0.05	0.06	0.05	0.16
**F_szm_2.5D.sze**	0.08	0.00	0.03	0.09	0.07	0.05
**F_szm_2.5D.lze**	0.67	0.53	0.80	0.84	0.81	0.46
**F_szm_2.5D.lgze**	0.48	0.42	0.21	0.10	0.30	0.14
**F_szm_2.5D.hgze**	0.07	0.05	0.05	0.02	0.14	0.02
**F_szm_2.5D.szlge**	0.59	0.49	0.21	0.18	0.41	0.15
**F_szm_2.5D.szhge**	0.06	0.03	0.08	0.08	0.11	0.06
**F_szm_2.5D.lzlge**	0.92	0.52	0.26	0.85	0.89	0.22
**F_szm_2.5D.lzhge**	0.06	0.50	0.91	0.90	0.72	0.52
**F_szm_2.5D.glnu**	0.43	0.35	0.29	0.17	0.26	0.38
**F_szm_2.5D.glnu.norm**	0.05	0.04	0.06	0.03	0.06	0.03

**F_szm_2.5D.zsnu**	0.34	0.36	0.26	0.04	0.24	0.27
**F_szm_2.5D.zsnu.norm**	0.15	0.01	0.07	0.16	0.14	0.11
**F_zsm_2.5D.z.perc**	0.10	0.10	0.16	0.33	0.24	0.12
**F_szm_2.5D.gl.var**	0.19	0.11	0.07	0.05	0.08	0.05
**F_szm_2.5D.zs.var**	0.86	0.60	1.04	0.87	0.92	0.69
**F_szm_2.5D.z.entr**	0.02	0.01	0.03	0.04	0.04	0.03
