# Supplementary material for: A Multicentre Evaluation of Dosiomics Features Reproducibility, Stability and Sensitivity
Source: Cancers (Basel). 2021 Jul 30;13(15):3835. doi: 10.3390/cancers13153835 (PMC8345157; doi:10.3390/cancers13153835)
Supplement: Supplementary file 1 [file cancers-13-03835-s001.zip › Table S2.pdf]

Table S2. Stability CV values for all the dosiomic features employed in the study and for all the six ROIs: left parotid, right parotid, PTV, Ring, Spinal Canal and Trachea.

Stability	Left Parotid	Right Parotid	PTV	RING	Spinal Canal	Trachea
F_stat.mean	0.13	0.03	0.01	0.06	0.09	0.16
F_stat.var	0.43	0.11	0.49	0.14	0.09	0.31
F_stat.skew	2.62	0.06	0.41	0.07	0.54	0.14
F_stat.kurt	4.71	0.99	0.64	4.02	0.48	0.41
F_stat.median	0.22	0.08	0.01	0.12	0.12	0.13
F_stat.min	0.23	0.16	0.04	0.08	0.15	0.08
F_stat.10thpercentile	0.27	0.16	0.01	0.05	0.28	0.09
F_stat.90thpercentile	0.06	0.04	0.01	0.09	0.05	0.20
F_stat.max	0.06	0.02	0.01	0.01	0.02	0.11
F_stat.iqr	0.32	0.11	0.32	0.10	0.08	0.23
F_stat.range	0.08	0.03	0.14	0.01	0.02	0.11
F_stat.mad	0.27	0.07	0.27	0.08	0.06	0.19
F_stat.rmad	0.33	0.10	0.32	0.09	0.08	0.23
F_stat.energy	0.62	0.50	0.47	0.56	0.50	0.67
F_stat.rms	0.10	0.03	0.01	0.06	0.07	0.17
F_stat.entropy	0.05	0.05	0.05	0.03	0.04	0.05
F_stat.uniformity	0.37	0.38	0.38	0.37	0.38	0.38
F_cm.joint.max	0.11	0.09	0.15	0.06	0.19	0.06
F_cm.joint.avg	0.14	0.03	0.01	0.07	0.10	0.16
F_cm.joint.var	0.41	0.18	0.22	0.19	0.20	0.35
F_cm.joint.entr	0.05	0.02	0.12	0.03	0.07	0.11
F_cm.diff.avg	0.29	0.18	0.24	0.19	0.29	0.19
F_cm.diff.var	0.29	0.39	0.22	0.31	0.38	0.17
F_cm.diff.entr	0.18	0.09	0.14	0.11	0.17	0.12
F_cm.sum.avg	0.14	0.03	0.01	0.07	0.10	0.16
F_cm.sum.var	0.41	0.18	0.24	0.19	0.20	0.36
F_cm.sum.entr	0.04	0.03	0.11	0.04	0.05	0.11
F_cm.energy	0.08	0.11	0.19	0.07	0.27	0.07
F_cm.contrast	0.35	0.36	0.28	0.33	0.42	0.22
F_cm.dissimilarity	0.29	0.18	0.24	0.19	0.29	0.19
F_cm.inv.diff	0.03	0.05	0.03	0.03	0.08	0.01
F_cm.inv.diff.norm	0.00	0.00	0.00	0.00	0.00	0.00
F_cm.inv.diff.mom	0.03	0.07	0.03	0.03	0.09	0.01
F_cm.inv.diff.mom.norm	0.00	0.00	0.00	0.00	0.00	0.00
F_cm.inv.var	0.24	0.04	0.21	0.05	0.15	0.16
F_cm.corr	0.02	0.01	0.09	0.00	0.01	0.03
F_cm.auto.corr	0.23	0.06	0.02	0.13	0.14	0.30
F_cm.clust.tend	0.41	0.18	0.24	0.19	0.20	0.36

**F_cm.clust.shade**	1.56	0.19	0.52	0.21	0.37	0.56
**F_cm.clust.prom**	0.74	0.27	0.51	0.23	0.30	0.54
**F_cm.info.corr.1**	0.08	0.04	0.18	0.06	0.08	0.07
**F_cm.info.corr.2**	0.09	0.00	0.07	0.04	0.01	0.09
**F_cm_merged.joint.max**	0.11	0.09	0.15	0.06	0.19	0.06
**F_cm_merged.joint.avg**	0.14	0.03	0.01	0.07	0.10	0.16
**F_cm_merged.joint.var**	0.41	0.18	0.22	0.19	0.20	0.35
**F_cm_merged.joint.entr**	0.06	0.02	0.12	0.03	0.08	0.11
**F_cm_merged.diff.avg**	0.29	0.18	0.24	0.19	0.29	0.19
**F_cm_merged.diff.var**	0.30	0.38	0.23	0.30	0.38	0.17
**F_cm_merged.diff.entr**	0.19	0.08	0.14	0.10	0.17	0.12
**F_cm_merged.sum.avg**	0.14	0.03	0.01	0.07	0.10	0.16
**F_cm_merged.sum.var**	0.41	0.18	0.24	0.19	0.20	0.36
**F_cm_merged.sum.entr**	0.04	0.02	0.11	0.04	0.05	0.11
**F_cm_merged.energy**	0.08	0.12	0.19	0.07	0.28	0.07
**F_cm_merged.contrast**	0.35	0.36	0.28	0.33	0.42	0.22
**F_cm_merged.dissimilarity**	0.29	0.18	0.24	0.19	0.29	0.19
**F_cm_merged.inv.diff**	0.03	0.05	0.03	0.03	0.08	0.01
**F_cm_merged.inv.diff.norm**	0.00	0.00	0.00	0.00	0.00	0.00
**F_cm_merged.inv.diff.mom**	0.03	0.07	0.03	0.03	0.09	0.01
**F_cm_merged.inv.diff.mom.norm**	0.00	0.00	0.00	0.00	0.00	0.00
**F_cm_merged.inv.var**	0.24	0.04	0.21	0.05	0.15	0.16
**F_cm_merged.corr**	0.01	0.01	0.08	0.00	0.01	0.03
**F_cm_merged.auto.corr**	0.23	0.06	0.02	0.13	0.14	0.30
**F_cm_merged.clust.tend**	0.41	0.18	0.24	0.19	0.20	0.36
**F_cm_merged.clust.shade**	1.56	0.19	0.52	0.21	0.37	0.56
**F_cm_merged.clust.prom**	0.74	0.27	0.51	0.23	0.30	0.54
**F_cm_merged.info.corr.1**	0.09	0.07	0.19	0.06	0.10	0.08
**F_cm_merged.info.corr.2**	0.10	0.00	0.06	0.04	0.01	0.10
**F_cm_2.5D.joint.max**	0.63	0.25	0.41	0.32	0.20	0.25
**F_cm_2.5D.joint.avg**	0.11	0.04	0.09	0.03	0.05	0.02
**F_cm_2.5D.joint.var**	0.17	0.06	0.30	0.04	0.09	0.04
**F_cm_2.5D.joint.entr**	0.03	0.02	0.04	0.04	0.03	0.02
**F_cm_2.5D.diff.avg**	0.21	0.20	0.22	0.22	0.25	0.20
**F_cm_2.5D.diff.var**	0.59	0.42	0.42	0.36	0.53	0.33
**F_cm_2.5D.diff.entr**	0.11	0.09	0.08	0.12	0.12	0.08
**F_cm_2.5D.sum.avg**	0.11	0.04	0.09	0.03	0.05	0.02
**F_cm_2.5D.sum.var**	0.17	0.07	0.32	0.04	0.09	0.04
**F_cm_2.5D.sum.entr**	0.01	0.01	0.04	0.00	0.01	0.00
**F_cm_2.5D.energy**	0.39	0.15	0.36	0.26	0.28	0.18
**F_cm_2.5D.contrast**	0.46	0.39	0.40	0.38	0.46	0.35
**F_cm_2.5D.dissimilarity**	0.21	0.20	0.22	0.22	0.25	0.20

**F_cm_2.5D.inv.diff**	0.10	0.09	0.15	0.11	0.14	0.13
**F_cm_2.5D.inv.diff.norm**	0.04	0.01	0.01	0.01	0.02	0.02
**F_cm_2.5D.inv.diff.mom**	0.15	0.12	0.22	0.15	0.21	0.20
**F_cm_2.5D.inv.diff.mom.norm**	0.03	0.00	0.00	0.00	0.00	0.01
**F_cm_2.5D.inv.var**	0.12	0.07	0.18	0.08	0.17	0.21
**F_cm_2.5D.corr**	0.01	0.00	0.05	0.00	0.01	0.01
**F_cm_2.5D.auto.corr**	0.12	0.07	0.14	0.05	0.09	0.04
**F_cm_2.5D.clust.tend**	0.17	0.07	0.32	0.04	0.09	0.04
**F_cm_2.5D.clust.shade**	0.71	0.05	0.16	0.22	0.39	0.42
**F_cm_2.5D.clust.prom**	0.29	0.05	0.35	0.05	0.14	0.06
**F_cm_2.5D.info.corr.1**	0.07	0.07	0.17	0.09	0.12	0.09
**F_cm_2.5D.info.corr.2**	0.00	0.00	0.00	0.00	0.00	0.00
**F_cm.2.5Dmerged.joint.max**	0.14	0.22	0.14	0.13	0.33	0.19
**F_cm.2.5Dmerged.joint.avg**	0.13	0.03	0.01	0.06	0.09	0.16
**F_cm.2.5Dmerged.joint.var**	0.42	0.11	0.51	0.14	0.10	0.31
**F_cm.2.5Dmerged.joint.entr**	0.11	0.02	0.14	0.03	0.05	0.08
**F_cm.2.5Dmerged.diff.avg**	0.29	0.18	0.23	0.19	0.29	0.18
**F_cm.2.5Dmerged.diff.var**	0.32	0.39	0.23	0.32	0.37	0.20
**F_cm.2.5Dmerged.diff.entr**	0.21	0.09	0.14	0.11	0.17	0.13
**F_cm.2.5Dmerged.sum.avg**	0.13	0.03	0.01	0.06	0.09	0.16
**F_cm.2.5Dmerged.sum.var**	0.43	0.11	0.52	0.14	0.10	0.31
**F_cm.2.5Dmerged.sum.entr**	0.08	0.01	0.13	0.02	0.02	0.08
**F_cm.2.5Dmerged.energy**	0.25	0.14	0.22	0.18	0.26	0.21
**F_cm.2.5Dmerged.contrast**	0.36	0.37	0.27	0.33	0.42	0.22
**F_cm.2.5Dmerged.dissimilarity**	0.29	0.18	0.23	0.19	0.29	0.18
**F_cm.2.5Dmerged.inv.diff**	0.03	0.06	0.02	0.03	0.08	0.01
**F_cm.2.5Dmerged.inv.diff.norm**	0.00	0.00	0.00	0.00	0.00	0.00
**F_cm.2.5Dmerged.inv.diff.mom**	0.03	0.07	0.02	0.04	0.09	0.01
**F_cm.2.5Dmerged.inv.diff.mom.norm**	0.00	0.00	0.00	0.00	0.00	0.00
**F_cm.2.5Dmerged.inv.var**	0.24	0.04	0.21	0.05	0.15	0.16
**F_cm.2.5Dmerged.corr**	0.01	0.00	0.04	0.00	0.00	0.00
**F_cm.2.5Dmerged.auto.corr**	0.21	0.06	0.02	0.13	0.14	0.30
**F_cm.2.5Dmerged.clust.tend**	0.43	0.11	0.52	0.14	0.10	0.31
**F_cm.2.5Dmerged.clust.shade**	1.45	0.13	0.66	0.15	0.48	0.43
**F_cm.2.5Dmerged.clust.prom**	0.71	0.16	0.64	0.18	0.18	0.54
**F_cm.2.5Dmerged.info.corr.1**	0.08	0.07	0.09	0.05	0.10	0.02
**F_cm.2.5Dmerged.info.corr.2**	0.12	0.00	0.07	0.00	0.00	0.01
**F_rlm.sre**	0.02	0.03	0.02	0.04	0.03	0.01
**F_rlm.lre**	0.17	0.22	0.18	0.30	0.27	0.11
**F_rlm.lgre**	0.40	0.14	0.38	0.15	0.17	0.20
**F_rlm.hgre**	0.09	0.09	0.13	0.03	0.08	0.04
**F_rlm.srlge**	0.34	0.29	0.35	0.15	0.19	0.21

**F_rlm.srhge**	0.09	0.09	0.12	0.05	0.08	0.03
**F_rlm.lrlge**	0.75	0.47	0.50	0.64	0.42	0.18
**F_rlm.lrhge**	0.12	0.18	0.28	0.24	0.21	0.15
**F_rlm.glnu**	0.31	0.30	0.39	0.37	0.37	0.42
**F_rlm.glnu.norm**	0.22	0.11	0.15	0.02	0.05	0.04
**F_rlm.rlnu**	0.47	0.37	0.39	0.29	0.35	0.42
**F_rlm.rlnu.norm**	0.05	0.06	0.04	0.09	0.07	0.02
**F_rlm.r.perc**	0.07	0.08	0.14	0.15	0.08	0.03
**F_rlm.gl.var**	0.13	0.07	0.18	0.03	0.09	0.05
**F_rlm.rl.var**	0.34	0.41	0.65	0.50	0.56	0.45
**F_rlm.rl.entr**	0.03	0.03	0.03	0.02	0.03	0.01
**F_rlm_merged.sre**	0.37	0.03	0.17	0.07	0.15	0.17
**F_rlm_merged.lre**	0.40	0.19	0.43	0.37	0.54	0.35
**F_rlm_merged.lgre**	0.30	0.31	0.02	0.07	0.42	0.14
**F_rlm_merged.hgre**	0.17	0.08	0.02	0.13	0.14	0.31
**F_rlm_merged.srlge**	0.48	0.27	0.18	0.17	0.33	0.18
**F_rlm_merged.srhge**	0.32	0.07	0.15	0.07	0.13	0.36
**F_rlm_merged.lrlge**	0.41	0.44	0.42	0.37	0.73	0.29
**F_rlm_merged.lrhge**	0.71	0.20	0.44	0.42	0.51	0.52
**F_rlm_merged.glnu**	0.29	0.26	0.23	0.25	0.25	0.24
**F_rlm_merged.glnu.norm**	0.10	0.15	0.09	0.08	0.18	0.09
**F_rlm_merged.rlnu**	0.23	0.35	0.21	0.21	0.12	0.37
**F_rlm_merged.rlnu.norm**	0.30	0.06	0.22	0.12	0.24	0.16
**F_rlm_merged.r.perc**	0.30	0.09	0.30	0.20	0.23	0.13
**F_rlm_merged.gl.var**	0.38	0.17	0.17	0.12	0.17	0.31
**F_rlm_merged.rl.var**	0.35	0.29	0.48	0.41	0.58	0.47
**F_rlm_merged.rl.entr**	0.06	0.04	0.07	0.05	0.05	0.06
**F_rlm_2.5D.sre**	0.38	0.03	0.16	0.07	0.15	0.17
**F_rlm_2.5D.lre**	0.46	0.21	0.44	0.29	0.58	0.36
**F_rlm_2.5D.lgre**	0.31	0.29	0.03	0.13	0.32	0.24
**F_rlm_2.5D.hgre**	0.15	0.08	0.03	0.11	0.13	0.32
**F_rlm_2.5D.srlge**	0.49	0.27	0.18	0.21	0.38	0.30
**F_rlm_2.5D.srhge**	0.36	0.06	0.15	0.04	0.11	0.36
**F_rlm_2.5D.lrhge**	0.73	0.22	0.45	0.44	0.59	0.53
**F_rlm_2.5D.glnu**	0.33	0.34	0.27	0.32	0.22	0.22
**F_rlm_2.5D.glnu.norm**	0.16	0.07	0.15	0.05	0.06	0.29
**F_rlm_2.5D.rlnu**	0.22	0.35	0.22	0.22	0.12	0.41
**F_rlm_2.5D.rlnu.norm**	0.29	0.06	0.23	0.12	0.25	0.19
**F_rlm_2.5D.gl.var**	0.35	0.11	0.28	0.09	0.11	0.29
**F_rlm_2.5D.rl.var**	0.38	0.30	0.49	0.31	0.61	0.38
**F_rlm_2.5D.rl.entr**	0.04	0.02	0.06	0.03	0.06	0.04
**F_rlm_2.5D.lrlrlm_25D_merged.dfge**	0.43	0.43	0.43	0.29	0.67	0.32

**F_rlm.2.5Dmerged.sre**	0.38	0.03	0.16	0.06	0.13	0.16
**F_rlm.2.5Dmerged.lre**	0.47	0.19	0.44	0.28	0.51	0.34
**F_rlm.2.5Dmerged.lgre**	0.31	0.29	0.03	0.13	0.32	0.24
**F_rlm.2.5Dmerged.hgre**	0.15	0.08	0.03	0.11	0.13	0.33
**F_rlm.2.5Dmerged.srlge**	0.49	0.26	0.18	0.21	0.36	0.30
**F_rlm.2.5Dmerged.srhge**	0.35	0.07	0.15	0.05	0.10	0.36
**F_rlm.2.5Dmerged.lrlge**	0.42	0.43	0.43	0.28	0.66	0.30
**F_rlm.2.5Dmerged.lrhge**	0.75	0.20	0.45	0.44	0.53	0.50
**F_rlm.2.5Dmerged.glnu**	0.33	0.34	0.27	0.31	0.22	0.21
**F_rlm.2.5Dmerged.glnu.norm**	0.16	0.07	0.16	0.06	0.06	0.29
**F_rlm.2.5Dmerged.rlnu**	0.22	0.35	0.22	0.22	0.12	0.40
**F_rlm.2.5Dmerged.rlnu.norm**	0.30	0.06	0.23	0.12	0.24	0.19
**F_rlm.2.5Dmerged.r.perc**	0.24	0.05	0.20	0.10	0.20	0.15
**F_rlm.2.5Dmerged.gl.var**	0.36	0.12	0.28	0.10	0.11	0.30
**F_rlm.2.5Dmerged.rl.var**	0.39	0.29	0.49	0.30	0.57	0.38
**F_rlm.2.5Dmerged.rl.entr**	0.04	0.02	0.05	0.03	0.07	0.04
**F_szm.sze**	1.03	0.04	0.16	0.09	0.21	0.24
**F_szm.lze**	0.95	0.50	0.83	0.74	0.85	0.76
**F_szm.lgze**	0.24	0.30	0.03	0.08	0.38	0.14
**F_szm.hgze**	0.17	0.07	0.03	0.07	0.10	0.29
**F_szm.szlge**	0.66	0.32	0.18	0.64	0.38	0.56
**F_szm.szhge**	1.20	0.06	0.15	0.08	0.23	0.41
**F_szm.lzlge**	1.07	0.51	0.82	0.73	0.83	0.72
**F_szm.lzhge**	1.19	0.50	0.84	0.76	1.07	0.89
**F_szm.glnu**	0.35	0.28	0.15	0.17	0.09	0.22
**F_szm.glnu.norm**	0.09	0.10	0.10	0.08	0.20	0.10
**F_szm.zsnu**	0.81	0.30	0.26	0.21	0.32	0.34
**F_szm.zsnu.norm**	0.35	0.08	0.18	0.08	0.25	0.11
**F_zsm.z.perc**	0.58	0.12	0.43	0.31	0.45	0.35
**F_szm.gl.var**	0.16	0.12	0.12	0.08	0.19	0.30
**F_szm.zs.var**	0.85	0.54	0.82	0.88	1.04	0.95
**F_szm.z.entr**	0.07	0.03	0.05	0.04	0.07	0.12
**F_szm_2.5D.sze**	0.09	0.01	0.03	0.07	0.07	0.04
**F_szm_2.5D.lze**	0.64	0.60	0.75	0.93	0.86	0.43
**F_szm_2.5D.lgze**	0.39	0.34	0.25	0.10	0.21	0.13
**F_szm_2.5D.hgze**	0.07	0.08	0.13	0.02	0.11	0.03
**F_szm_2.5D.szlge**	0.51	0.44	0.22	0.18	0.30	0.13
**F_szm_2.5D.szhge**	0.08	0.06	0.12	0.07	0.09	0.05
**F_szm_2.5D.lzlge**	1.19	0.71	0.60	1.00	0.82	0.24
**F_szm_2.5D.lzhge**	0.17	0.44	0.84	0.93	0.73	0.53
**F_szm_2.5D.glnu**	0.43	0.31	0.28	0.16	0.24	0.37
**F_szm_2.5D.glnu.norm**	0.05	0.05	0.17	0.02	0.05	0.02

**F_szm_2.5D.zsnu**	0.30	0.34	0.28	0.03	0.20	0.29
**F_szm_2.5D.zsnu.norm**	0.18	0.03	0.07	0.13	0.13	0.08
**F_zsm_2.5D.z.perc**	0.13	0.10	0.14	0.28	0.22	0.10
**F_szm_2.5D.gl.var**	0.16	0.08	0.18	0.04	0.09	0.04
**F_szm_2.5D.zs.var**	0.81	0.68	1.00	0.96	1.00	0.70
**F_szm_2.5D.z.entr**	0.03	0.01	0.03	0.03	0.04	0.02
