# Supplementary material for: A Multicentre Evaluation of Dosiomics Features Reproducibility, Stability and Sensitivity
Source: Cancers (Basel). 2021 Jul 30;13(15):3835. doi: 10.3390/cancers13153835 (PMC8345157; doi:10.3390/cancers13153835)
Supplement: Supplementary file 1 [file cancers-13-03835-s001.zip › Table S3.pdf]

Table S3. Sensitivity (1 mm dose calculation grid) CV values for all the dosiomic features employed in the study and for all the six ROIs: left parotid, right parotid, PTV, Ring, Spinal Canal and Trachea.

Sensitivity 1 mm	Left Parotid	Right Parotid	PTV	RING	Spinal Canal	Trachea
F_stat.mean	0.40	0.29	0.28	0.22	0.40	0.69
F_stat.var	2.18	0.29	2.95	0.25	0.66	1.36
F_stat.skew	1.39	0.43	2.04	0.25	5.38	0.44
F_stat.kurt	2.37	0.81	1.56	0.93	4.49	1.44
F_stat.median	0.46	0.37	0.31	0.49	0.48	0.53
F_stat.min	0.61	0.66	0.33	0.40	0.72	0.42
F_stat.10thpercentile	0.64	0.52	0.32	0.39	0.79	0.43
F_stat.90thpercentile	0.53	0.20	0.20	0.18	0.34	0.79
F_stat.max	0.69	0.10	0.10	0.10	0.34	0.64
F_stat.iqr	1.01	0.20	1.63	0.22	0.49	0.97
F_stat.range	0.86	0.12	0.98	0.10	0.36	0.65
F_stat.mad	0.98	0.16	1.61	0.15	0.43	0.85
F_stat.rmad	1.04	0.17	1.69	0.18	0.48	0.94
F_stat.energy	1.19	0.33	0.31	0.42	0.51	1.30
F_stat.rms	0.43	0.24	0.26	0.17	0.36	0.73
F_stat.entropy	0.03	0.03	0.01	0.08	0.00	0.00
F_stat.uniformity	0.16	0.16	0.10	2.32	0.04	0.01
F_cm.joint.max	0.31	1.41	0.38	0.12	0.85	0.21
F_cm.joint.avg	0.40	0.29	0.28	0.20	0.40	0.69
F_cm.joint.var	1.10	0.44	1.28	0.43	0.50	1.15
F_cm.joint.entr	0.23	0.20	0.34	0.15	0.25	0.38
F_cm.diff.avg	0.33	0.32	0.52	0.22	0.37	0.55
F_cm.diff.var	0.24	0.43	0.52	0.43	0.44	0.46
F_cm.diff.entr	0.19	0.24	0.31	0.16	0.25	0.36
F_cm.sum.avg	0.40	0.29	0.28	0.20	0.40	0.69
F_cm.sum.var	1.12	0.44	1.36	0.43	0.50	1.16
F_cm.sum.entr	0.23	0.19	0.30	0.14	0.24	0.38
F_cm.energy	0.46	2.27	0.50	0.15	1.01	0.24
F_cm.contrast	0.33	0.44	0.70	0.37	0.52	0.62
F_cm.dissimilarity	0.33	0.32	0.52	0.22	0.37	0.55
F_cm.inv.diff	0.03	0.21	0.08	0.03	0.08	0.03
F_cm.inv.diff.norm	0.00	0.01	0.00	0.00	0.00	0.00
F_cm.inv.diff.mom	0.03	0.26	0.08	0.04	0.08	0.03
F_cm.inv.diff.mom.norm	0.00	0.00	0.00	0.00	0.00	0.00
F_cm.inv.var	0.33	0.20	0.42	0.12	0.29	0.51
F_cm.corr	0.05	0.02	0.10	0.03	0.02	0.06
F_cm.auto.corr	0.78	0.38	0.32	0.31	0.50	1.30
F_cm.clust.tend	1.12	0.44	1.36	0.43	0.50	1.16

**F_cm.clust.shade**	1.94	0.65	4.30	0.59	0.74	1.76
**F_cm.clust.prom**	1.74	0.74	2.78	0.65	0.79	1.84
**F_cm.info.corr.1**	0.05	0.03	0.27	0.04	0.04	0.08
**F_cm.info.corr.2**	0.13	0.07	0.18	0.08	0.10	0.22
**F_cm_merged.joint.max**	0.31	1.44	0.39	0.12	0.85	0.21
**F_cm_merged.joint.avg**	0.40	0.29	0.28	0.20	0.40	0.69
**F_cm_merged.joint.var**	1.10	0.44	1.28	0.43	0.50	1.15
**F_cm_merged.joint.entr**	0.24	0.21	0.34	0.15	0.25	0.39
**F_cm_merged.diff.avg**	0.33	0.32	0.53	0.22	0.37	0.55
**F_cm_merged.diff.var**	0.27	0.43	0.52	0.42	0.42	0.50
**F_cm_merged.diff.entr**	0.21	0.24	0.31	0.15	0.24	0.38
**F_cm_merged.sum.avg**	0.40	0.29	0.28	0.20	0.40	0.69
**F_cm_merged.sum.var**	1.12	0.44	1.36	0.43	0.50	1.16
**F_cm_merged.sum.entr**	0.23	0.19	0.30	0.13	0.24	0.38
**F_cm_merged.energy**	0.47	2.41	0.50	0.15	1.03	0.24
**F_cm_merged.contrast**	0.33	0.44	0.70	0.37	0.52	0.62
**F_cm_merged.dissimilarity**	0.33	0.32	0.53	0.22	0.37	0.55
**F_cm_merged.inv.diff**	0.03	0.21	0.08	0.03	0.08	0.03
**F_cm_merged.inv.diff.norm**	0.00	0.01	0.00	0.00	0.00	0.00
**F_cm_merged.inv.diff.mom**	0.03	0.26	0.08	0.04	0.08	0.03
**F_cm_merged.inv.diff.mom.norm**	0.00	0.00	0.00	0.00	0.00	0.00
**F_cm_merged.inv.var**	0.33	0.20	0.42	0.12	0.29	0.51
**F_cm_merged.corr**	0.04	0.02	0.10	0.03	0.02	0.06
**F_cm_merged.auto.corr**	0.78	0.38	0.32	0.31	0.50	1.30
**F_cm_merged.clust.tend**	1.12	0.44	1.36	0.43	0.50	1.16
**F_cm_merged.clust.shade**	1.94	0.65	4.30	0.59	0.75	1.76
**F_cm_merged.clust.prom**	1.74	0.74	2.78	0.65	0.79	1.84
**F_cm_merged.info.corr.1**	0.03	0.07	0.28	0.04	0.04	0.08
**F_cm_merged.info.corr.2**	0.13	0.07	0.08	0.08	0.10	0.22
**F_cm_2.5D.joint.max**	2.24	0.57	0.46	1.87	2.91	1.69
**F_cm_2.5D.joint.avg**	0.18	0.14	0.22	0.14	0.25	0.16
**F_cm_2.5D.joint.var**	0.45	0.13	0.34	0.11	0.17	0.07
**F_cm_2.5D.joint.entr**	0.06	0.03	0.04	0.04	0.13	0.03
**F_cm_2.5D.diff.avg**	0.10	0.06	0.22	0.29	0.11	0.12
**F_cm_2.5D.diff.var**	0.19	0.16	0.32	0.46	0.75	0.44
**F_cm_2.5D.diff.entr**	0.05	0.03	0.09	0.10	0.10	0.07
**F_cm_2.5D.sum.avg**	0.18	0.14	0.22	0.14	0.25	0.16
**F_cm_2.5D.sum.var**	0.46	0.13	0.37	0.11	0.17	0.07
**F_cm_2.5D.sum.entr**	0.03	0.02	0.03	0.02	0.11	0.02
**F_cm_2.5D.energy**	1.52	0.27	0.38	0.92	2.92	0.87
**F_cm_2.5D.contrast**	0.12	0.08	0.34	0.59	0.28	0.30
**F_cm_2.5D.dissimilarity**	0.10	0.06	0.22	0.29	0.11	0.12

**F_cm_2.5D.inv.diff**	0.15	0.06	0.16	0.12	0.24	0.09
**F_cm_2.5D.inv.diff.norm**	0.06	0.01	0.01	0.01	0.03	0.09
**F_cm_2.5D.inv.diff.mom**	0.24	0.10	0.24	0.16	0.38	0.15
**F_cm_2.5D.inv.diff.mom.norm**	0.05	0.00	0.00	0.00	0.04	0.07
**F_cm_2.5D.inv.var**	0.19	0.09	0.23	0.15	0.11	0.09
**F_cm_2.5D.corr**	0.00	0.00	0.06	0.00	0.01	0.01
**F_cm_2.5D.auto.corr**	0.34	0.20	0.34	0.20	0.31	0.22
**F_cm_2.5D.clust.tend**	0.46	0.13	0.37	0.11	0.17	0.07
**F_cm_2.5D.clust.shade**	0.62	0.36	2.60	0.43	0.37	2.14
**F_cm_2.5D.clust.prom**	0.70	0.19	0.55	0.22	0.15	0.09
**F_cm_2.5D.info.corr.1**	0.15	0.07	0.27	0.07	0.13	0.09
**F_cm_2.5D.info.corr.2**	0.00	0.00	0.00	0.00	0.00	0.00
**F_cm.2.5Dmerged.joint.max**	0.47	1.41	0.50	0.35	1.39	0.47
**F_cm.2.5Dmerged.joint.avg**	0.41	0.29	0.28	0.22	0.40	0.69
**F_cm.2.5Dmerged.joint.var**	2.18	0.29	2.94	0.25	0.67	1.36
**F_cm.2.5Dmerged.joint.entr**	0.23	0.13	0.35	0.09	0.24	0.35
**F_cm.2.5Dmerged.diff.avg**	0.32	0.32	0.52	0.24	0.37	0.55
**F_cm.2.5Dmerged.diff.var**	0.27	0.44	0.51	0.39	0.41	0.55
**F_cm.2.5Dmerged.diff.entr**	0.20	0.23	0.29	0.16	0.23	0.38
**F_cm.2.5Dmerged.sum.avg**	0.41	0.29	0.28	0.22	0.40	0.69
**F_cm.2.5Dmerged.sum.var**	2.19	0.29	2.97	0.25	0.67	1.36
**F_cm.2.5Dmerged.sum.entr**	0.23	0.09	0.35	0.08	0.23	0.34
**F_cm.2.5Dmerged.energy**	0.66	1.99	0.65	0.42	1.90	0.68
**F_cm.2.5Dmerged.contrast**	0.32	0.44	0.68	0.38	0.52	0.62
**F_cm.2.5Dmerged.dissimilarity**	0.32	0.32	0.52	0.24	0.37	0.55
**F_cm.2.5Dmerged.inv.diff**	0.03	0.21	0.08	0.05	0.08	0.03
**F_cm.2.5Dmerged.inv.diff.norm**	0.00	0.01	0.00	0.00	0.00	0.00
**F_cm.2.5Dmerged.inv.diff.mom**	0.03	0.27	0.08	0.06	0.08	0.03
**F_cm.2.5Dmerged.inv.diff.mom.norm**	0.00	0.00	0.00	0.00	0.00	0.00
**F_cm.2.5Dmerged.inv.var**	0.32	0.20	0.42	0.15	0.29	0.50
**F_cm.2.5Dmerged.corr**	0.02	0.00	0.10	0.00	0.02	0.01
**F_cm.2.5Dmerged.auto.corr**	0.77	0.37	0.32	0.33	0.50	1.29
**F_cm.2.5Dmerged.clust.tend**	2.19	0.29	2.97	0.25	0.67	1.36
**F_cm.2.5Dmerged.clust.shade**	3.26	0.61	3.33	0.29	7.89	1.58
**F_cm.2.5Dmerged.clust.prom**	3.23	0.57	3.31	0.33	1.28	1.78
**F_cm.2.5Dmerged.info.corr.1**	0.06	0.16	0.31	0.05	0.08	0.04
**F_cm.2.5Dmerged.info.corr.2**	0.10	0.00	0.10	0.00	0.09	0.23
**F_rlm.sre**	0.06	0.01	0.01	0.02	0.04	0.02
**F_rlm.lre**	0.83	0.08	0.08	0.30	1.24	0.16
**F_rlm.lgre**	0.87	0.32	0.39	0.98	1.55	0.87
**F_rlm.hgre**	0.34	0.17	0.31	0.16	0.22	0.19
**F_rlm.srlge**	0.30	0.26	0.36	0.43	0.46	0.43

**F_rlm.srhge**	0.26	0.17	0.30	0.16	0.21	0.19
**F_rlm.lrlge**	3.01	0.55	0.56	2.89	3.23	2.71
**F_rlm.lrhge**	0.92	0.28	0.37	0.25	0.25	0.22
**F_rlm.glnu**	0.12	0.11	0.11	0.29	0.13	0.07
**F_rlm.glnu.norm**	0.16	0.14	0.17	0.08	0.37	0.10
**F_rlm.rlnu**	0.17	0.14	0.12	0.28	0.21	0.06
**F_rlm.rlnu.norm**	0.07	0.03	0.03	0.06	0.09	0.04
**F_rlm.r.perc**	0.07	0.02	0.06	0.04	0.13	0.03
**F_rlm.gl.var**	0.34	0.12	0.30	0.13	0.06	0.06
**F_rlm.rl.var**	1.64	0.25	0.57	0.68	2.53	0.68
**F_rlm.rl.entr**	0.02	0.01	0.03	0.01	0.02	0.01
**F_rlm_merged.sre**	0.35	0.24	0.26	0.17	0.25	0.40
**F_rlm_merged.lre**	0.64	2.54	0.92	0.40	1.03	0.31
**F_rlm_merged.lgre**	1.69	2.87	3.29	0.19	1.60	0.29
**F_rlm_merged.hgre**	0.78	0.36	0.32	0.26	0.49	1.34
**F_rlm_merged.srlge**	1.13	1.59	3.17	0.46	1.08	0.19
**F_rlm_merged.srhge**	1.32	0.36	0.40	0.28	0.50	1.62
**F_rlm_merged.lrlge**	2.09	3.30	3.31	0.49	2.12	0.39
**F_rlm_merged.lrhge**	0.65	0.36	0.96	0.35	0.52	0.63
**F_rlm_merged.glnu**	0.17	0.49	0.20	0.23	0.18	0.08
**F_rlm_merged.glnu.norm**	0.38	1.63	0.24	0.14	0.59	0.21
**F_rlm_merged.rlnu**	0.64	0.30	0.70	0.37	0.48	0.80
**F_rlm_merged.rlnu.norm**	0.39	0.26	0.37	0.19	0.30	0.35
**F_rlm_merged.r.perc**	0.24	0.24	0.39	0.18	0.29	0.34
**F_rlm_merged.gl.var**	0.83	0.43	1.15	0.41	0.46	1.13
**F_rlm_merged.rl.var**	0.52	2.44	0.85	0.31	1.10	0.21
**F_rlm_merged.rl.entr**	0.03	0.04	0.07	0.05	0.05	0.04
**F_rlm_2.5D.sre**	0.30	0.19	0.21	0.16	0.23	0.47
**F_rlm_2.5D.lre**	0.49	2.04	0.85	0.25	1.10	0.44
**F_rlm_2.5D.lgre**	1.87	2.52	3.25	0.80	1.67	0.51
**F_rlm_2.5D.hgre**	1.14	0.31	0.30	0.25	0.45	1.34
**F_rlm_2.5D.srlge**	1.33	1.40	3.05	0.85	0.97	0.37
**F_rlm_2.5D.srhge**	1.87	0.32	0.38	0.27	0.46	1.59
**F_rlm_2.5D.lrhge**	0.60	0.31	0.97	0.30	0.59	0.74
**F_rlm_2.5D.glnu**	0.21	0.10	0.34	0.34	0.30	0.40
**F_rlm_2.5D.glnu.norm**	0.50	0.26	0.41	0.26	0.66	0.63
**F_rlm_2.5D.rlnu**	0.63	0.29	0.72	0.39	0.50	0.83
**F_rlm_2.5D.rlnu.norm**	0.31	0.25	0.36	0.22	0.30	0.47
**F_rlm_2.5D.gl.var**	2.38	0.27	2.93	0.16	0.88	1.28
**F_rlm_2.5D.rl.var**	0.53	2.46	0.86	0.28	1.23	0.31
**F_rlm_2.5D.rl.entr**	0.10	0.06	0.18	0.02	0.11	0.09
**F_rlm_2.5D.lrlrlm_25D_merged.dfge**	2.28	3.29	3.30	0.42	2.30	0.69

**F_rlm.2.5Dmerged.sre**	0.36	0.18	0.21	0.15	0.21	0.47
**F_rlm.2.5Dmerged.lre**	0.56	2.04	0.84	0.25	1.08	0.43
**F_rlm.2.5Dmerged.lgre**	1.88	2.50	3.25	0.77	1.65	0.52
**F_rlm.2.5Dmerged.hgre**	1.15	0.31	0.30	0.24	0.45	1.35
**F_rlm.2.5Dmerged.srlge**	1.33	1.39	3.04	0.82	0.92	0.39
**F_rlm.2.5Dmerged.srhge**	1.86	0.32	0.38	0.27	0.47	1.60
**F_rlm.2.5Dmerged.lrlge**	2.28	3.29	3.30	0.42	2.32	0.69
**F_rlm.2.5Dmerged.lrhge**	0.68	0.31	0.96	0.30	0.51	0.73
**F_rlm.2.5Dmerged.glnu**	0.21	0.09	0.34	0.34	0.30	0.41
**F_rlm.2.5Dmerged.glnu.norm**	0.51	0.23	0.42	0.22	0.65	0.64
**F_rlm.2.5Dmerged.rlnu**	0.64	0.30	0.73	0.39	0.53	0.82
**F_rlm.2.5Dmerged.rlnu.norm**	0.37	0.26	0.37	0.23	0.30	0.48
**F_rlm.2.5Dmerged.r.perc**	0.23	0.23	0.37	0.16	0.28	0.33
**F_rlm.2.5Dmerged.gl.var**	2.42	0.26	2.95	0.16	0.91	1.30
**F_rlm.2.5Dmerged.rl.var**	0.54	2.49	0.85	0.28	1.23	0.32
**F_rlm.2.5Dmerged.rl.entr**	0.10	0.07	0.19	0.02	0.10	0.08
**F_szm.sze**	0.44	0.29	0.32	0.25	0.74	0.58
**F_szm.lze**	1.47	3.30	1.82	0.51	1.91	0.35
**F_szm.lgze**	1.44	3.04	3.28	0.19	1.50	0.28
**F_szm.hgze**	0.87	0.34	0.31	0.23	0.45	1.42
**F_szm.szlge**	1.83	1.08	2.63	0.54	1.24	1.17
**F_szm.szhge**	2.16	0.34	0.37	0.26	0.77	1.84
**F_szm.lzlge**	2.37	3.32	3.32	0.63	2.68	0.38
**F_szm.lzhge**	0.83	1.97	1.10	0.66	0.65	0.40
**F_szm.glnu**	0.13	0.26	0.37	0.33	0.62	0.15
**F_szm.glnu.norm**	0.36	1.62	0.32	0.15	0.56	0.22
**F_szm.zsnu**	0.27	0.48	0.68	0.50	1.78	0.41
**F_szm.zsnu.norm**	0.36	0.17	0.29	0.12	0.57	0.22
**F_zsm.z.perc**	0.23	0.36	0.68	0.35	0.87	0.65
**F_szm.gl.var**	0.69	0.43	1.03	0.39	0.36	1.03
**F_szm.zs.var**	0.92	3.25	0.91	0.54	1.38	0.43
**F_szm.z.entr**	0.13	0.19	0.22	0.16	0.20	0.31
**F_szm_2.5D.sze**	0.24	0.05	0.03	0.08	0.03	0.02
**F_szm_2.5D.lze**	2.05	0.26	0.33	0.92	2.92	0.98
**F_szm_2.5D.lgze**	0.29	0.15	0.27	0.30	0.32	0.20
**F_szm_2.5D.hgze**	0.36	0.11	0.29	0.08	0.14	0.14
**F_szm_2.5D.szlge**	0.40	0.27	0.14	0.50	0.35	0.16
**F_szm_2.5D.szhge**	0.18	0.09	0.26	0.08	0.11	0.12
**F_szm_2.5D.lzlge**	3.25	0.80	0.95	3.12	3.31	3.20
**F_szm_2.5D.lzhge**	1.86	0.52	0.60	0.61	0.28	0.26
**F_szm_2.5D.glnu**	0.33	0.28	0.11	0.31	0.14	0.03
**F_szm_2.5D.glnu.norm**	0.11	0.06	0.15	0.04	0.12	0.05

**F_szm_2.5D.zsnu**	0.51	0.39	0.14	0.32	0.17	0.07
**F_szm_2.5D.zsnu.norm**	0.26	0.10	0.07	0.16	0.06	0.04
**F_zsm_2.5D.z.perc**	0.23	0.08	0.08	0.22	0.17	0.05
**F_szm_2.5D.gl.var**	0.23	0.07	0.23	0.09	0.07	0.06
**F_szm_2.5D.zs.var**	2.47	0.36	0.71	1.07	3.16	1.78
**F_szm_2.5D.z.entr**	0.04	0.02	0.03	0.03	0.02	0.01
