# Supplementary material for: A Multicentre Evaluation of Dosiomics Features Reproducibility, Stability and Sensitivity
Source: Cancers (Basel). 2021 Jul 30;13(15):3835. doi: 10.3390/cancers13153835 (PMC8345157; doi:10.3390/cancers13153835)
Supplement: Supplementary file 1 [file cancers-13-03835-s001.zip › Table S4.pdf]

**Table S4.** Sensitivity (2 mm dose calculation grid) CV values for all the dosiomic features employed in the study and for all the six ROIs: left parotid, right parotid, PTV, Ring, Spinal Canal and Trachea.

| Sensitivity 2 mm | Left Parotid | Right Parotid | PTV | RING | Spinal Canal | Trachea |
|---|---|---|---|---|---|---|
| F_stat.mean | 0.39 | 0.15 | 0.02 | 0.17 | 0.36 | 0.61 |
| F_stat.var | 0.91 | 0.22 | 0.82 | 0.14 | 0.52 | 1.38 |
| F_stat.skew | 1.64 | 0.37 | 1.50 | 0.24 | 71.34 | 0.31 |
| F_stat.kurt | 3.63 | 0.49 | 1.35 | 0.85 | 4.58 | 0.90 |
| F_stat.median | 0.40 | 0.21 | 0.02 | 0.37 | 0.38 | 0.50 |
| F_stat.min | 0.57 | 0.50 | 0.05 | 0.38 | 0.64 | 0.42 |
| F_stat.10thpercentile | 0.55 | 0.38 | 0.01 | 0.40 | 0.64 | 0.42 |
| F_stat.90thpercentile | 0.33 | 0.05 | 0.02 | 0.13 | 0.33 | 0.70 |
| F_stat.max | 0.27 | 0.03 | 0.03 | 0.02 | 0.30 | 0.51 |
| F_stat.iqr | 0.69 | 0.15 | 0.33 | 0.17 | 0.42 | 0.92 |
| F_stat.range | 0.26 | 0.10 | 0.51 | 0.03 | 0.31 | 0.52 |
| F_stat.mad | 0.50 | 0.11 | 0.33 | 0.09 | 0.37 | 0.76 |
| F_stat.rmad | 0.64 | 0.13 | 0.32 | 0.13 | 0.41 | 0.88 |
| F_stat.energy | 0.62 | 0.21 | 0.03 | 0.23 | 0.46 | 1.30 |
| F_stat.rms | 0.36 | 0.11 | 0.02 | 0.11 | 0.35 | 0.64 |
| F_stat.entropy | 0.01 | 0.01 | 0.02 | 0.02 | 0.01 | 0.02 |
| F_stat.uniformity | 0.19 | 0.19 | 0.34 | 0.89 | 0.18 | 0.30 |
| F_cm.joint.max | 0.32 | 0.18 | 0.30 | 0.13 | 0.89 | 0.17 |
| F_cm.joint.avg | 0.38 | 0.16 | 0.02 | 0.17 | 0.37 | 0.61 |
| F_cm.joint.var | 1.36 | 0.32 | 0.97 | 0.32 | 0.44 | 1.05 |
| F_cm.joint.entr | 0.24 | 0.04 | 0.31 | 0.10 | 0.21 | 0.33 |
| F_cm.diff.avg | 0.38 | 0.15 | 0.45 | 0.15 | 0.31 | 0.48 |
| F_cm.diff.var | 0.26 | 0.37 | 0.47 | 0.39 | 0.40 | 0.40 |
| F_cm.diff.entr | 0.20 | 0.08 | 0.26 | 0.13 | 0.21 | 0.32 |
| F_cm.sum.avg | 0.38 | 0.16 | 0.02 | 0.17 | 0.37 | 0.61 |
| F_cm.sum.var | 1.38 | 0.32 | 1.02 | 0.32 | 0.44 | 1.06 |
| F_cm.sum.entr | 0.23 | 0.03 | 0.28 | 0.10 | 0.20 | 0.32 |
| F_cm.energy | 0.47 | 0.26 | 0.38 | 0.16 | 1.09 | 0.20 |
| F_cm.contrast | 0.38 | 0.33 | 0.61 | 0.31 | 0.46 | 0.54 |
| F_cm.dissimilarity | 0.38 | 0.15 | 0.45 | 0.15 | 0.31 | 0.48 |
| F_cm.inv.diff | 0.03 | 0.06 | 0.06 | 0.02 | 0.06 | 0.02 |
| F_cm.inv.diff.norm | 0.00 | 0.00 | 0.00 | 0.00 | 0.00 | 0.00 |
| F_cm.inv.diff.mom | 0.03 | 0.08 | 0.06 | 0.03 | 0.07 | 0.02 |
| F_cm.inv.diff.mom.norm | 0.00 | 0.00 | 0.00 | 0.00 | 0.00 | 0.00 |
| F_cm.inv.var | 0.38 | 0.08 | 0.35 | 0.10 | 0.23 | 0.44 |
| F_cm.corr | 0.05 | 0.00 | 0.08 | 0.02 | 0.02 | 0.07 |
| F_cm.auto.corr | 0.60 | 0.22 | 0.03 | 0.23 | 0.47 | 1.30 |
| F_cm.clust.tend | 1.38 | 0.32 | 1.02 | 0.32 | 0.44 | 1.06 |

| | | | | | | |
|---|---|---|---|---|---|---|
| **F_cm.clust.shade** | 1.72 | 0.54 | 2.12 | 0.52 | 0.72 | 1.97 |
| **F_cm.clust.prom** | 2.17 | 0.72 | 2.47 | 0.63 | 0.77 | 2.18 |
| **F_cm.info.corr.1** | 0.05 | 0.05 | 0.13 | 0.02 | 0.04 | 0.08 |
| **F_cm.info.corr.2** | 0.18 | 0.00 | 0.15 | 0.08 | 0.09 | 0.19 |
| **F_cm_merged.joint.max** | 0.32 | 0.18 | 0.30 | 0.13 | 0.89 | 0.17 |
| **F_cm_merged.joint.avg** | 0.38 | 0.16 | 0.02 | 0.17 | 0.37 | 0.61 |
| **F_cm_merged.joint.var** | 1.36 | 0.32 | 0.97 | 0.32 | 0.44 | 1.05 |
| **F_cm_merged.joint.entr** | 0.25 | 0.04 | 0.31 | 0.11 | 0.21 | 0.33 |
| **F_cm_merged.diff.avg** | 0.38 | 0.15 | 0.45 | 0.15 | 0.31 | 0.48 |
| **F_cm_merged.diff.var** | 0.29 | 0.36 | 0.47 | 0.38 | 0.38 | 0.43 |
| **F_cm_merged.diff.entr** | 0.23 | 0.07 | 0.26 | 0.13 | 0.21 | 0.33 |
| **F_cm_merged.sum.avg** | 0.38 | 0.16 | 0.02 | 0.17 | 0.37 | 0.61 |
| **F_cm_merged.sum.var** | 1.38 | 0.32 | 1.02 | 0.32 | 0.44 | 1.06 |
| **F_cm_merged.sum.entr** | 0.24 | 0.03 | 0.28 | 0.10 | 0.20 | 0.32 |
| **F_cm_merged.energy** | 0.47 | 0.30 | 0.38 | 0.16 | 1.11 | 0.20 |
| **F_cm_merged.contrast** | 0.38 | 0.33 | 0.61 | 0.31 | 0.46 | 0.54 |
| **F_cm_merged.dissimilarity** | 0.38 | 0.15 | 0.45 | 0.15 | 0.31 | 0.48 |
| **F_cm_merged.inv.diff** | 0.03 | 0.06 | 0.06 | 0.02 | 0.06 | 0.02 |
| **F_cm_merged.inv.diff.norm** | 0.00 | 0.00 | 0.00 | 0.00 | 0.00 | 0.00 |
| **F_cm_merged.inv.diff.mom** | 0.03 | 0.08 | 0.06 | 0.03 | 0.07 | 0.02 |
| **F_cm_merged.inv.diff.mom.norm** | 0.00 | 0.00 | 0.00 | 0.00 | 0.00 | 0.00 |
| **F_cm_merged.inv.var** | 0.38 | 0.08 | 0.35 | 0.10 | 0.23 | 0.44 |
| **F_cm_merged.corr** | 0.05 | 0.00 | 0.08 | 0.02 | 0.01 | 0.07 |
| **F_cm_merged.auto.corr** | 0.60 | 0.22 | 0.03 | 0.23 | 0.47 | 1.30 |
| **F_cm_merged.clust.tend** | 1.38 | 0.32 | 1.02 | 0.32 | 0.44 | 1.06 |
| **F_cm_merged.clust.shade** | 1.72 | 0.54 | 2.12 | 0.52 | 0.72 | 1.97 |
| **F_cm_merged.clust.prom** | 2.17 | 0.72 | 2.47 | 0.63 | 0.77 | 2.18 |
| **F_cm_merged.info.corr.1** | 0.03 | 0.04 | 0.14 | 0.03 | 0.04 | 0.08 |
| **F_cm_merged.info.corr.2** | 0.19 | 0.00 | 0.09 | 0.07 | 0.09 | 0.19 |
| **F_cm_2.5D.joint.max** | 2.23 | 0.33 | 0.84 | 1.79 | 2.86 | 1.80 |
| **F_cm_2.5D.joint.avg** | 0.18 | 0.13 | 0.21 | 0.13 | 0.23 | 0.14 |
| **F_cm_2.5D.joint.var** | 0.47 | 0.11 | 0.28 | 0.11 | 0.13 | 0.04 |
| **F_cm_2.5D.joint.entr** | 0.06 | 0.02 | 0.06 | 0.02 | 0.12 | 0.03 |
| **F_cm_2.5D.diff.avg** | 0.08 | 0.06 | 0.13 | 0.05 | 0.08 | 0.10 |
| **F_cm_2.5D.diff.var** | 0.47 | 0.28 | 0.20 | 0.34 | 0.89 | 0.39 |
| **F_cm_2.5D.diff.entr** | 0.06 | 0.03 | 0.05 | 0.04 | 0.10 | 0.07 |
| **F_cm_2.5D.sum.avg** | 0.18 | 0.13 | 0.21 | 0.13 | 0.23 | 0.14 |
| **F_cm_2.5D.sum.var** | 0.47 | 0.11 | 0.30 | 0.11 | 0.13 | 0.04 |
| **F_cm_2.5D.sum.entr** | 0.03 | 0.01 | 0.04 | 0.02 | 0.10 | 0.01 |
| **F_cm_2.5D.energy** | 1.51 | 0.19 | 0.65 | 0.84 | 2.85 | 0.84 |
| **F_cm_2.5D.contrast** | 0.18 | 0.15 | 0.18 | 0.20 | 0.32 | 0.24 |
| **F_cm_2.5D.dissimilarity** | 0.08 | 0.06 | 0.13 | 0.05 | 0.08 | 0.10 |

| | | | | | | |
|---|---|---|---|---|---|---|
| **F_cm_2.5D.inv.diff** | 0.16 | 0.06 | 0.18 | 0.04 | 0.23 | 0.10 |
| **F_cm_2.5D.inv.diff.norm** | 0.04 | 0.00 | 0.01 | 0.00 | 0.03 | 0.06 |
| **F_cm_2.5D.inv.diff.mom** | 0.26 | 0.09 | 0.29 | 0.06 | 0.36 | 0.17 |
| **F_cm_2.5D.inv.diff.mom.norm** | 0.04 | 0.00 | 0.00 | 0.00 | 0.04 | 0.05 |
| **F_cm_2.5D.inv.var** | 0.19 | 0.09 | 0.19 | 0.09 | 0.10 | 0.08 |
| **F_cm_2.5D.corr** | 0.01 | 0.00 | 0.05 | 0.00 | 0.01 | 0.00 |
| **F_cm_2.5D.auto.corr** | 0.34 | 0.19 | 0.33 | 0.18 | 0.28 | 0.19 |
| **F_cm_2.5D.clust.tend** | 0.47 | 0.11 | 0.30 | 0.11 | 0.13 | 0.04 |
| **F_cm_2.5D.clust.shade** | 0.74 | 0.24 | 1.31 | 0.36 | 0.31 | 2.11 |
| **F_cm_2.5D.clust.prom** | 0.72 | 0.11 | 0.36 | 0.22 | 0.11 | 0.08 |
| **F_cm_2.5D.info.corr.1** | 0.14 | 0.06 | 0.12 | 0.02 | 0.12 | 0.09 |
| **F_cm_2.5D.info.corr.2** | 0.00 | 0.00 | 0.00 | 0.00 | 0.00 | 0.00 |
| **F_cm.2.5Dmerged.joint.max** | 0.46 | 0.33 | 0.33 | 0.38 | 1.54 | 0.41 |
| **F_cm.2.5Dmerged.joint.avg** | 0.39 | 0.15 | 0.02 | 0.17 | 0.37 | 0.61 |
| **F_cm.2.5Dmerged.joint.var** | 0.92 | 0.22 | 0.75 | 0.14 | 0.52 | 1.38 |
| **F_cm.2.5Dmerged.joint.entr** | 0.20 | 0.03 | 0.28 | 0.06 | 0.23 | 0.30 |
| **F_cm.2.5Dmerged.diff.avg** | 0.36 | 0.15 | 0.44 | 0.15 | 0.31 | 0.48 |
| **F_cm.2.5Dmerged.diff.var** | 0.28 | 0.36 | 0.47 | 0.32 | 0.37 | 0.48 |
| **F_cm.2.5Dmerged.diff.entr** | 0.22 | 0.07 | 0.26 | 0.08 | 0.20 | 0.33 |
| **F_cm.2.5Dmerged.sum.avg** | 0.39 | 0.15 | 0.02 | 0.17 | 0.37 | 0.61 |
| **F_cm.2.5Dmerged.sum.var** | 0.92 | 0.22 | 0.77 | 0.14 | 0.52 | 1.38 |
| **F_cm.2.5Dmerged.sum.entr** | 0.20 | 0.02 | 0.26 | 0.06 | 0.23 | 0.29 |
| **F_cm.2.5Dmerged.energy** | 0.62 | 0.14 | 0.44 | 0.45 | 2.04 | 0.59 |
| **F_cm.2.5Dmerged.contrast** | 0.36 | 0.33 | 0.59 | 0.30 | 0.46 | 0.54 |
| **F_cm.2.5Dmerged.dissimilarity** | 0.36 | 0.15 | 0.44 | 0.15 | 0.31 | 0.48 |
| **F_cm.2.5Dmerged.inv.diff** | 0.03 | 0.06 | 0.05 | 0.03 | 0.07 | 0.02 |
| **F_cm.2.5Dmerged.inv.diff.norm** | 0.00 | 0.00 | 0.00 | 0.00 | 0.00 | 0.00 |
| **F_cm.2.5Dmerged.inv.diff.mom** | 0.03 | 0.08 | 0.06 | 0.03 | 0.07 | 0.02 |
| **F_cm.2.5Dmerged.inv.diff.mom.norm** | 0.00 | 0.00 | 0.00 | 0.00 | 0.00 | 0.00 |
| **F_cm.2.5Dmerged.inv.var** | 0.36 | 0.08 | 0.35 | 0.10 | 0.23 | 0.44 |
| **F_cm.2.5Dmerged.corr** | 0.01 | 0.00 | 0.06 | 0.00 | 0.02 | 0.01 |
| **F_cm.2.5Dmerged.auto.corr** | 0.62 | 0.21 | 0.03 | 0.23 | 0.46 | 1.30 |
| **F_cm.2.5Dmerged.clust.tend** | 0.92 | 0.22 | 0.77 | 0.14 | 0.52 | 1.38 |
| **F_cm.2.5Dmerged.clust.shade** | 2.90 | 0.61 | 2.49 | 0.19 | 1.77 | 1.74 |
| **F_cm.2.5Dmerged.clust.prom** | 1.44 | 0.54 | 2.31 | 0.18 | 0.73 | 2.16 |
| **F_cm.2.5Dmerged.info.corr.1** | 0.04 | 0.06 | 0.15 | 0.04 | 0.06 | 0.03 |
| **F_cm.2.5Dmerged.info.corr.2** | 0.10 | 0.00 | 0.10 | 0.00 | 0.10 | 0.14 |
| **F_rlm.sre** | 0.07 | 0.03 | 0.04 | 0.04 | 0.05 | 0.03 |
| **F_rlm.lre** | 0.85 | 0.10 | 0.19 | 0.26 | 1.18 | 0.15 |
| **F_rlm.lgre** | 0.85 | 0.25 | 0.32 | 1.01 | 1.47 | 0.83 |
| **F_rlm.hgre** | 0.35 | 0.16 | 0.29 | 0.15 | 0.19 | 0.17 |
| **F_rlm.srlge** | 0.27 | 0.19 | 0.30 | 0.44 | 0.39 | 0.35 |

| | | | | | | |
|---|---|---|---|---|---|---|
| **F_rlm.srhge** | 0.27 | 0.16 | 0.27 | 0.14 | 0.19 | 0.17 |
| **F_rlm.lrlge** | 2.99 | 0.45 | 0.41 | 2.89 | 3.22 | 2.63 |
| **F_rlm.lrhge** | 0.93 | 0.23 | 0.45 | 0.25 | 0.23 | 0.19 |
| **F_rlm.glnu** | 0.11 | 0.09 | 0.13 | 0.09 | 0.13 | 0.06 |
| **F_rlm.glnu.norm** | 0.16 | 0.11 | 0.18 | 0.07 | 0.36 | 0.08 |
| **F_rlm.rlnu** | 0.14 | 0.09 | 0.12 | 0.12 | 0.21 | 0.10 |
| **F_rlm.rlnu.norm** | 0.10 | 0.07 | 0.08 | 0.08 | 0.11 | 0.07 |
| **F_rlm.r.perc** | 0.08 | 0.03 | 0.05 | 0.05 | 0.13 | 0.04 |
| **F_rlm.gl.var** | 0.34 | 0.10 | 0.19 | 0.13 | 0.04 | 0.05 |
| **F_rlm.rl.var** | 1.67 | 0.21 | 0.69 | 0.59 | 2.47 | 0.50 |
| **F_rlm.rl.entr** | 0.02 | 0.01 | 0.02 | 0.02 | 0.02 | 0.01 |
| **F_rlm_merged.sre** | 0.41 | 0.05 | 0.18 | 0.13 | 0.18 | 0.36 |
| **F_rlm_merged.lre** | 0.54 | 0.14 | 0.60 | 0.30 | 0.88 | 0.26 |
| **F_rlm_merged.lgre** | 2.07 | 1.72 | 0.03 | 0.19 | 0.99 | 0.25 |
| **F_rlm_merged.hgre** | 0.57 | 0.19 | 0.03 | 0.17 | 0.45 | 1.35 |
| **F_rlm_merged.srlge** | 1.29 | 1.75 | 0.16 | 0.23 | 0.59 | 0.23 |
| **F_rlm_merged.srhge** | 0.51 | 0.19 | 0.19 | 0.16 | 0.46 | 1.82 |
| **F_rlm_merged.lrlge** | 2.89 | 1.49 | 0.64 | 0.41 | 1.32 | 0.35 |
| **F_rlm_merged.lrhge** | 0.71 | 0.32 | 0.56 | 0.34 | 0.50 | 0.57 |
| **F_rlm_merged.glnu** | 0.12 | 0.08 | 0.13 | 0.06 | 0.06 | 0.07 |
| **F_rlm_merged.glnu.norm** | 0.36 | 0.13 | 0.23 | 0.15 | 0.34 | 0.18 |
| **F_rlm_merged.rlnu** | 0.76 | 0.13 | 0.60 | 0.20 | 0.43 | 0.71 |
| **F_rlm_merged.rlnu.norm** | 0.44 | 0.09 | 0.29 | 0.15 | 0.24 | 0.29 |
| **F_rlm_merged.r.perc** | 0.25 | 0.05 | 0.30 | 0.10 | 0.22 | 0.28 |
| **F_rlm_merged.gl.var** | 0.96 | 0.30 | 0.80 | 0.29 | 0.41 | 1.05 |
| **F_rlm_merged.rl.var** | 0.48 | 0.25 | 0.61 | 0.20 | 1.23 | 0.19 |
| **F_rlm_merged.rl.entr** | 0.03 | 0.01 | 0.04 | 0.01 | 0.05 | 0.03 |
| **F_rlm_2.5D.sre** | 0.35 | 0.05 | 0.18 | 0.07 | 0.20 | 0.42 |
| **F_rlm_2.5D.lre** | 0.45 | 0.12 | 0.61 | 0.25 | 1.16 | 0.36 |
| **F_rlm_2.5D.lgre** | 2.14 | 1.82 | 0.03 | 0.28 | 0.68 | 0.42 |
| **F_rlm_2.5D.hgre** | 0.58 | 0.18 | 0.03 | 0.14 | 0.44 | 1.33 |
| **F_rlm_2.5D.srlge** | 1.51 | 1.80 | 0.17 | 0.16 | 0.47 | 0.29 |
| **F_rlm_2.5D.srhge** | 0.52 | 0.17 | 0.20 | 0.14 | 0.45 | 1.76 |
| **F_rlm_2.5D.lrhge** | 0.64 | 0.31 | 0.58 | 0.28 | 0.57 | 0.65 |
| **F_rlm_2.5D.glnu** | 0.17 | 0.06 | 0.13 | 0.10 | 0.19 | 0.35 |
| **F_rlm_2.5D.glnu.norm** | 0.41 | 0.07 | 0.24 | 0.08 | 0.64 | 0.54 |
| **F_rlm_2.5D.rlnu** | 0.71 | 0.12 | 0.59 | 0.21 | 0.40 | 0.76 |
| **F_rlm_2.5D.rlnu.norm** | 0.34 | 0.09 | 0.29 | 0.12 | 0.24 | 0.40 |
| **F_rlm_2.5D.gl.var** | 0.65 | 0.25 | 0.75 | 0.11 | 0.45 | 1.24 |
| **F_rlm_2.5D.rl.var** | 0.47 | 0.22 | 0.62 | 0.27 | 1.38 | 0.25 |
| **F_rlm_2.5D.rl.entr** | 0.05 | 0.02 | 0.06 | 0.02 | 0.08 | 0.07 |
| **F_rlm_2.5D.lrlrlm_25D_merged.dfge** | 2.77 | 1.61 | 0.65 | 0.43 | 1.21 | 0.58 |

| | | | | | | |
|---|---|---|---|---|---|---|
| **F_rlm.2.5Dmerged.sre** | 0.42 | 0.04 | 0.18 | 0.07 | 0.18 | 0.42 |
| **F_rlm.2.5Dmerged.lre** | 0.53 | 0.11 | 0.61 | 0.25 | 1.16 | 0.36 |
| **F_rlm.2.5Dmerged.lgre** | 2.15 | 1.83 | 0.03 | 0.28 | 0.68 | 0.42 |
| **F_rlm.2.5Dmerged.hgre** | 0.58 | 0.18 | 0.03 | 0.14 | 0.44 | 1.34 |
| **F_rlm.2.5Dmerged.srlge** | 1.50 | 1.82 | 0.16 | 0.16 | 0.45 | 0.30 |
| **F_rlm.2.5Dmerged.srhge** | 0.55 | 0.17 | 0.19 | 0.14 | 0.46 | 1.75 |
| **F_rlm.2.5Dmerged.lrlge** | 2.78 | 1.61 | 0.65 | 0.43 | 1.22 | 0.58 |
| **F_rlm.2.5Dmerged.lrhge** | 0.71 | 0.31 | 0.57 | 0.28 | 0.49 | 0.65 |
| **F_rlm.2.5Dmerged.glnu** | 0.17 | 0.06 | 0.13 | 0.10 | 0.19 | 0.36 |
| **F_rlm.2.5Dmerged.glnu.norm** | 0.42 | 0.07 | 0.24 | 0.09 | 0.62 | 0.54 |
| **F_rlm.2.5Dmerged.rlnu** | 0.73 | 0.13 | 0.60 | 0.21 | 0.44 | 0.76 |
| **F_rlm.2.5Dmerged.rlnu.norm** | 0.42 | 0.09 | 0.29 | 0.12 | 0.25 | 0.41 |
| **F_rlm.2.5Dmerged.r.perc** | 0.25 | 0.05 | 0.30 | 0.10 | 0.22 | 0.28 |
| **F_rlm.2.5Dmerged.gl.var** | 0.61 | 0.25 | 0.75 | 0.11 | 0.45 | 1.25 |
| **F_rlm.2.5Dmerged.rl.var** | 0.48 | 0.22 | 0.61 | 0.27 | 1.38 | 0.26 |
| **F_rlm.2.5Dmerged.rl.entr** | 0.06 | 0.02 | 0.06 | 0.02 | 0.07 | 0.07 |
| **F_szm.sze** | 0.51 | 0.17 | 0.25 | 0.20 | 0.81 | 0.59 |
| **F_szm.lze** | 0.69 | 0.40 | 0.90 | 0.40 | 0.99 | 0.30 |
| **F_szm.lgze** | 1.70 | 1.36 | 0.03 | 0.18 | 1.02 | 0.25 |
| **F_szm.hgze** | 0.54 | 0.19 | 0.03 | 0.11 | 0.44 | 1.47 |
| **F_szm.szlge** | 1.89 | 1.19 | 0.25 | 0.44 | 0.86 | 0.87 |
| **F_szm.szhge** | 0.72 | 0.21 | 0.25 | 0.18 | 0.85 | 2.26 |
| **F_szm.lzlge** | 2.98 | 1.47 | 0.95 | 0.55 | 1.46 | 0.36 |
| **F_szm.lzhge** | 0.91 | 0.51 | 0.85 | 0.61 | 0.60 | 0.40 |
| **F_szm.glnu** | 0.13 | 0.14 | 0.29 | 0.16 | 0.63 | 0.14 |
| **F_szm.glnu.norm** | 0.34 | 0.09 | 0.24 | 0.15 | 0.34 | 0.19 |
| **F_szm.zsnu** | 0.07 | 0.43 | 0.73 | 0.42 | 1.73 | 0.28 |
| **F_szm.zsnu.norm** | 0.26 | 0.24 | 0.19 | 0.13 | 0.39 | 0.20 |
| **F_zsm.z.perc** | 0.28 | 0.20 | 0.58 | 0.26 | 0.81 | 0.55 |
| **F_szm.gl.var** | 0.73 | 0.30 | 0.69 | 0.27 | 0.33 | 0.95 |
| **F_szm.zs.var** | 0.90 | 0.46 | 0.76 | 0.41 | 1.52 | 0.48 |
| **F_szm.z.entr** | 0.15 | 0.02 | 0.16 | 0.09 | 0.15 | 0.27 |
| **F_szm_2.5D.sze** | 0.27 | 0.12 | 0.10 | 0.13 | 0.11 | 0.11 |
| **F_szm_2.5D.lze** | 2.03 | 0.21 | 0.70 | 0.76 | 2.87 | 0.75 |
| **F_szm_2.5D.lgze** | 0.28 | 0.19 | 0.25 | 0.20 | 0.28 | 0.17 |
| **F_szm_2.5D.hgze** | 0.38 | 0.09 | 0.27 | 0.08 | 0.11 | 0.12 |
| **F_szm_2.5D.szlge** | 0.41 | 0.32 | 0.21 | 0.24 | 0.38 | 0.24 |
| **F_szm_2.5D.szhge** | 0.25 | 0.15 | 0.25 | 0.14 | 0.13 | 0.16 |
| **F_szm_2.5D.lzlge** | 3.25 | 0.70 | 0.59 | 3.12 | 3.31 | 3.15 |
| **F_szm_2.5D.lzhge** | 1.70 | 0.34 | 0.93 | 0.48 | 0.34 | 0.29 |
| **F_szm_2.5D.glnu** | 0.22 | 0.10 | 0.13 | 0.09 | 0.15 | 0.11 |
| **F_szm_2.5D.glnu.norm** | 0.11 | 0.04 | 0.15 | 0.02 | 0.11 | 0.04 |

| | | | | | | |
|---|---|---|---|---|---|---|
| **F_szm_2.5D.zsnu** | 0.43 | 0.27 | 0.25 | 0.21 | 0.29 | 0.21 |
| **F_szm_2.5D.zsnu.norm** | 0.30 | 0.18 | 0.16 | 0.17 | 0.16 | 0.16 |
| **F_zsm_2.5D.z.perc** | 0.26 | 0.12 | 0.14 | 0.08 | 0.20 | 0.10 |
| **F_szm_2.5D.gl.var** | 0.22 | 0.06 | 0.15 | 0.08 | 0.05 | 0.05 |
| **F_szm_2.5D.zs.var** | 2.48 | 0.20 | 1.04 | 0.91 | 3.14 | 1.44 |
| **F_szm_2.5D.z.entr** | 0.05 | 0.03 | 0.03 | 0.03 | 0.04 | 0.03 |
