# Supplementary material for: A Multicentre Evaluation of Dosiomics Features Reproducibility, Stability and Sensitivity
Source: Cancers (Basel). 2021 Jul 30;13(15):3835. doi: 10.3390/cancers13153835 (PMC8345157; doi:10.3390/cancers13153835)
Supplement: Supplementary file 1 [file cancers-13-03835-s001.zip › Table S5.pdf]

Table S5. mean values of the different CV for all the studies, ROIs and family's features.

		Sensitivity 1 mm	Sensitivity 2 mm	Stability	Reproducibility
**RING**					
	STAT	0,403	0,229	0,351	0,326
	CM	0,224	0,173	0,111	0,127
	RLM	0,337	0,227	0,183	0,189
	SZM	0,429	0,351	0,312	0,306
**Left Parotid**					
	STAT	0,880	0,740	0,639	0,653
	CM	0,506	0,492	0,239	0,265
	RLM	0,805	0,729	0,302	0,322
	SZM	0,853	0,789	0,468	0,487
**Right Parotid**					
	STAT	0,299	0,194	0,171	0,149
	CM	0,330	0,158	0,107	0,113
	RLM	0,769	0,356	0,182	0,182
	SZM	0,751	0,326	0,255	0,252
**PTV**					
	STAT	0,865	0,323	0,212	0,228
	CM	0,643	0,398	0,180	0,180
	RLM	0,904	0,276	0,233	0,241
	SZM	0,689	0,341	0,320	0,303
**Spinal Canal**					
	STAT	0,957	1,463	0,181	0,207
	CM	0,474	0,395	0,160	0,148
	RLM	0,722	0,565	0,258	0,267
	SZM	0,864	0,744	0,369	0,368
**Trachea**					
	STAT	0,717	0,538	0,216	0,272
	CM	0,516	0,373	0,160	0,208
	RLM	0,553	0,415	0,243	0,287
	SZM	0,531	0,439	0,303	0,326
**ALL**					
		0,626	0,460	0,257	0,267
	STAT	0,687	0,581	0,295	0,306
	CM	0,449	0,331	0,160	0,173
	RLM	0,682	0,428	0,233	0,248
	SZM	0,686	0,498	0,338	0,340
