# Supplementary material for: A Multicentre Evaluation of Dosiomics Features Reproducibility, Stability and Sensitivity
Source: Cancers (Basel). 2021 Jul 30;13(15):3835. doi: 10.3390/cancers13153835 (PMC8345157; doi:10.3390/cancers13153835)
Supplement: Supplementary file 1 [file cancers-13-03835-s001.zip › Table S9.pdf]

Table S9. common dosiomic features between the following studies and relative threshold: reproducibility (CV_{TH})<0.3) and stability (CV_{TH})<0.3), sensitivity 1 mm (CV_{TH})>1) and sensitivity 2 mm (CV_{TH})>1), stability (CV_{TH} <0.3) and sensitivity 1 mm (CV_{TH})>1), stability (CV_{TH})<0.3) and sensitivity 1 mm (CV_{TH})>1) for the ROI PTV. Abbreviation: Rep.= reproducibility; Stab.= stability; Sens.= sensitivity.

**PTV**	**Repr. (CV_{TH}) <0.3) ∩ Stab. (CV_{TH} <0.3)**	**Sens. 1 mm (CV_{TH} >1) ∩ Sens. 2 mm (CV_{TH} >1)**	**Stab. (CV_{TH} < 0.3) ∩ Sens. 1 mm (CV_{TH} >1)**	**Stab. (CV_{TH} < 0.3) ∩ Sens. 2 mm (CV_{TH} >1)**
F_stat.10thpercentile	X			
F_stat.90thpercentile	X			
F_stat.entropy	X			
F_stat.kurt		X		
F_stat.mad			X	
F_stat.max	X			
F_stat.mean	X			
F_stat.median	X			
F_stat.min	X			
F_stat.range	X			
F_stat.rms	X			
F_stat.skew		X		
F_cm_2.5D.inv.diff.norm	X			
F_cm_2.5D.inv.var	X			
F_cm_2.5D.joint.avg	X			
F_cm_2.5D.joint.entr	X			
F_cm_2.5D.sum.avg	X			
F_cm_2.5D.sum.entr	X			
F_cm_merged.auto.corr	X			
F_cm_merged.clust.prom		X		
F_cm_merged.clust.shade		X		
F_cm_merged.clust.tend	X	X	X	X
F_cm_merged.corr	X			
F_cm_merged.diff.entr	X			
F_cm_merged.diff.var	X			
F_cm_merged.energy	X			
F_cm_merged.info.corr.1	X			
F_cm_merged.info.corr.2	X			
F_cm_merged.inv.diff	X			
F_cm_merged.inv.diff.mom	X			
F_cm_merged.inv.diff.mom.norm	X			
F_cm_merged.inv.diff.norm	X			
F_cm_merged.inv.var	X			
F_cm_merged.joint.avg	X			

F_cm_merged.joint.entr	X			
F_cm_merged.joint.max	X			
F_cm_merged.joint.var	X		X	
F_cm_merged.sum.avg	X			
F_cm_merged.sum.entr	X			
F_cm_merged.sum.var	X	X	X	X
F_cm.2.5Dmerged.auto.corr	X			
F_cm.2.5Dmerged.clust.prom		X		
F_cm.2.5Dmerged.clust.shade		X		
F_cm.2.5Dmerged.corr	X			
F_cm.2.5Dmerged.diff.avg	X			
F_cm.2.5Dmerged.diff.entr	X			
F_cm.2.5Dmerged.diff.var	X			
F_cm.2.5Dmerged.dissimilarity	X			
F_cm.2.5Dmerged.energy	X			
F_cm.2.5Dmerged.info.corr.1	X			
F_cm.2.5Dmerged.info.corr.2	X			
F_cm.2.5Dmerged.inv.diff	X			
F_cm.2.5Dmerged.inv.diff.mom	X			
F_cm.2.5Dmerged.inv.diff.mom.norm	X			
F_cm.2.5Dmerged.inv.diff.norm	X			
F_cm.2.5Dmerged.inv.var	X			
F_cm.2.5Dmerged.joint.avg	X			
F_cm.2.5Dmerged.joint.entr	X			
F_cm.2.5Dmerged.joint.max	X			
F_cm.2.5Dmerged.sum.avg	X			
F_cm.2.5Dmerged.sum.entr	X			
F_cm.auto.corr	X			
F_cm.clust.prom		X		
F_cm.clust.shade		X		
F_cm.clust.tend	X	X	X	X
F_cm.corr	X			
F_cm.diff.entr	X			
F_cm.diff.var	X			
F_cm.energy	X			
F_cm.info.corr.1	X			
F_cm.info.corr.2	X			
F_cm.inv.diff	X			
F_cm.inv.diff.mom	X			
F_cm.inv.diff.mom.norm	X			
F_cm.inv.diff.norm	X			
F_cm.inv.var	X			
F_cm.joint.avg	X			
F_cm.joint.entr	X			

F_cm.joint.max	X			
F_cm.joint.var	X		X	
F_cm.sum.avg	X			
F_cm.sum.entr	X			
F_cm.sum.var	X	X	X	X
F_rlm_2.5D.gl.var			X	
F_rlm_2.5D.glnu.norm	X			
F_rlm_2.5D.hgre	X			
F_rlm_2.5D.lgre	X		X	
F_rlm_2.5D.rl.entr	X			
F_rlm_2.5D.rlnu	X			
F_rlm_2.5D.rlnu.norm	X			
F_rlm_2.5D.sre	X			
F_rlm_2.5D.srhge	X			
F_rlm_2.5D.srlge	X		X	
F_rlm_merged.gl.var	X		X	
F_rlm_merged.glnu	X			
F_rlm_merged.glnu.norm	X			
F_rlm_merged.hgre	X			
F_rlm_merged.lgre	X		X	
F_rlm_merged.rl.entr	X			
F_rlm_merged.rlnu	X			
F_rlm_merged.rlnu.norm	X			
F_rlm_merged.sre	X			
F_rlm_merged.srhge	X			
F_rlm_merged.srlge	X		X	
F_rlm.2.5Dmerged.gl.var			X	
F_rlm.2.5Dmerged.glnu.norm	X			
F_rlm.2.5Dmerged.hgre	X			
F_rlm.2.5Dmerged.lgre	X		X	
F_rlm.2.5Dmerged.r.perc	X			
F_rlm.2.5Dmerged.rl.entr	X			
F_rlm.2.5Dmerged.rlnu	X			
F_rlm.2.5Dmerged.rlnu.norm	X			
F_rlm.2.5Dmerged.sre	X			
F_rlm.2.5Dmerged.srhge	X			
F_rlm.2.5Dmerged.srlge	X		X	
F_rlm.gl.var	X			
F_rlm.glnu.norm	X			
F_rlm.hgre	X			
F_rlm.lre	X			
F_rlm.r.perc	X			
F_rlm.rl.entr	X			
F_rlm.rlnu.norm	X			

F_rlm.sre	X	
F_rlm.srhge	X	
F_szm_2.5D.gl.var	X	
F_szm_2.5D.glnu	X	
F_szm_2.5D.glnu.norm	X	
F_szm_2.5D.hgze	X	
F_szm_2.5D.lgze	X	
F_szm_2.5D.sze	X	
F_szm_2.5D.szhge	X	
F_szm_2.5D.szlge	X	
F_szm_2.5D.z.entr	X	
F_szm_2.5D.zsnu	X	
F_szm_2.5D.zsnu.norm	X	
F_szm.gl.var	X	X
F_szm.gl.var		X
F_szm.glnu	X	
F_szm.glnu.norm	X	
F_szm.hgze	X	
F_szm.lgze	X	X
F_szm.sze	X	
F_szm.szhge	X	
F_szm.szlge	X	X
F_szm.z.entr	X	
F_szm.zsnu	X	
F_szm.zsnu.norm	X	
F_zsm_2.5D.z.perc	X	
