# Supplementary figures and images for: A Multicentre Evaluation of Dosiomics Features Reproducibility, Stability and Sensitivity
Source: Cancers (Basel). 2021 Jul 30;13(15):3835. doi: 10.3390/cancers13153835 (PMC8345157; doi:10.3390/cancers13153835)

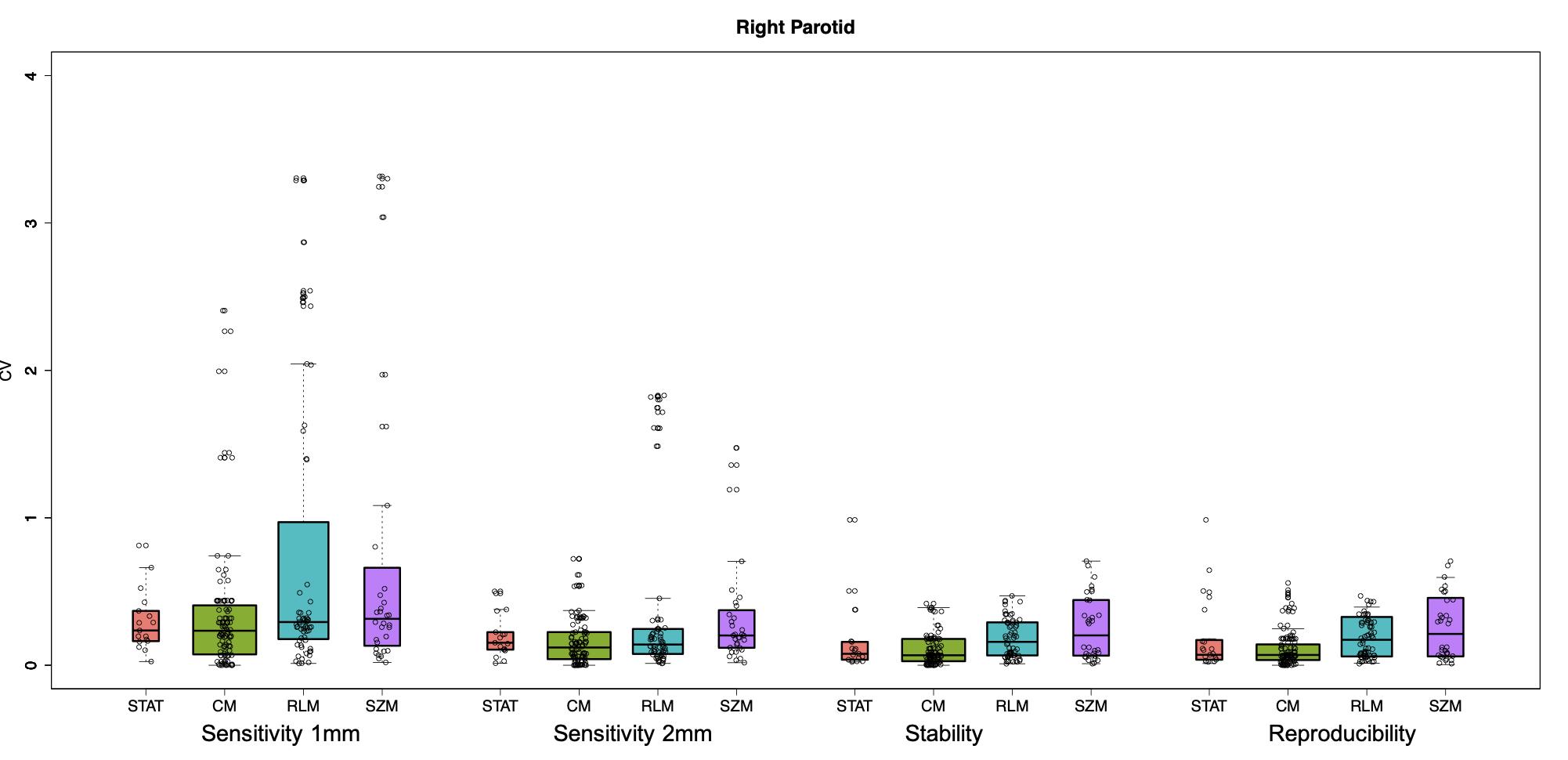

Supplement: Supplementary file 1 [file cancers-13-03835-s001.zip › Figure S1.jpg]

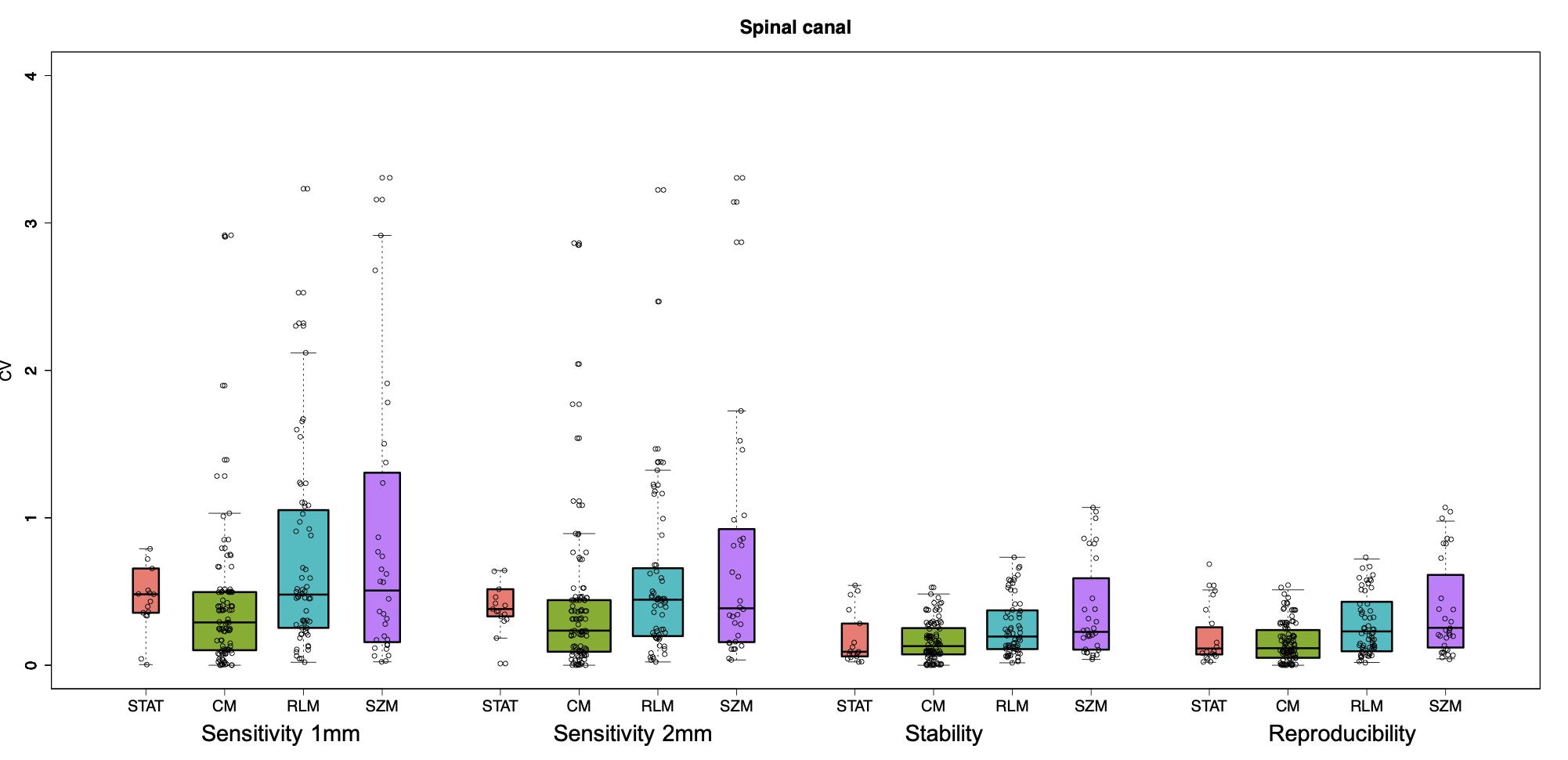

Supplement: Supplementary file 1 [file cancers-13-03835-s001.zip › Figure S2.jpg]

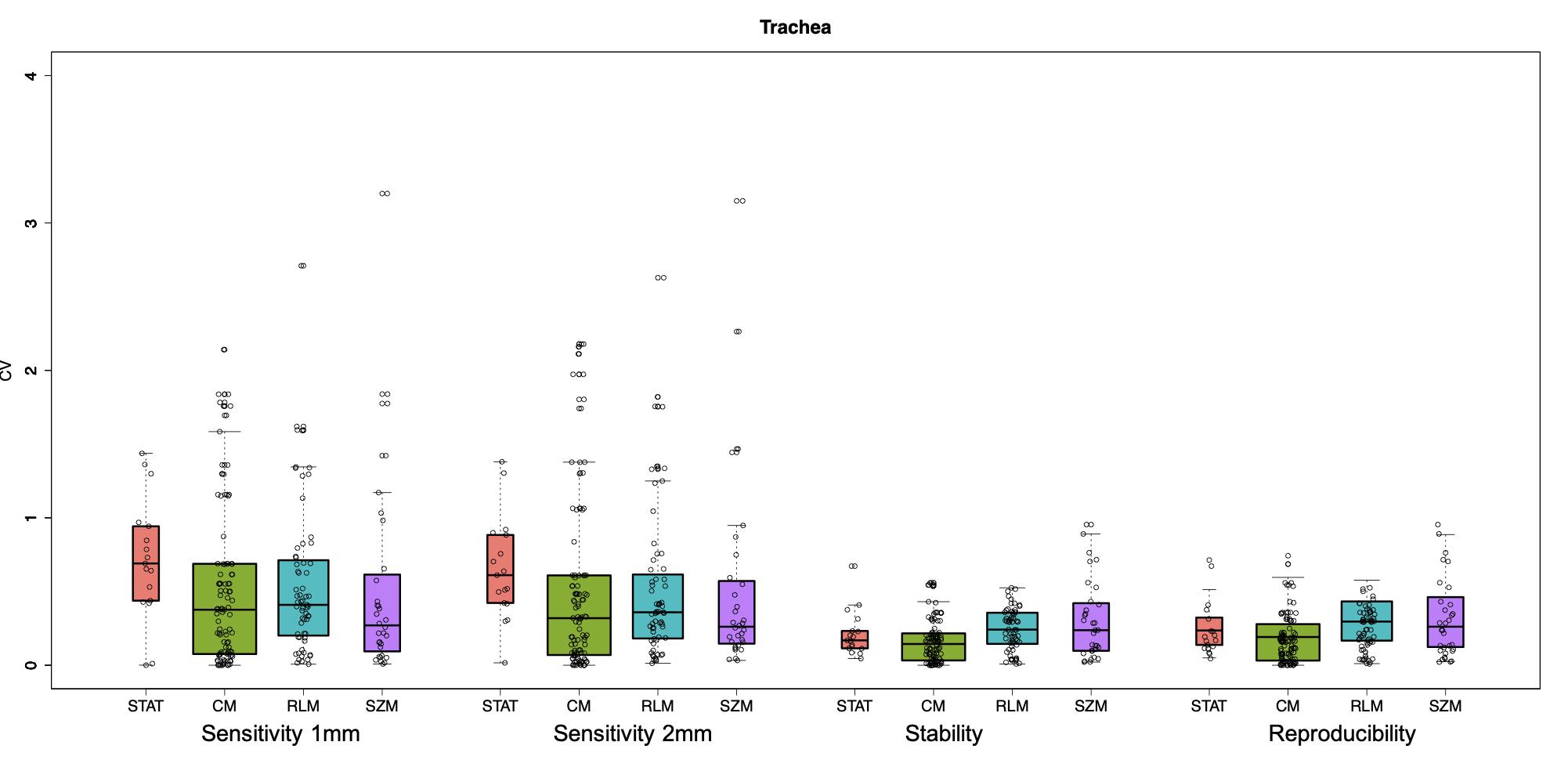

Supplement: Supplementary file 1 [file cancers-13-03835-s001.zip › Figure S3.jpg]

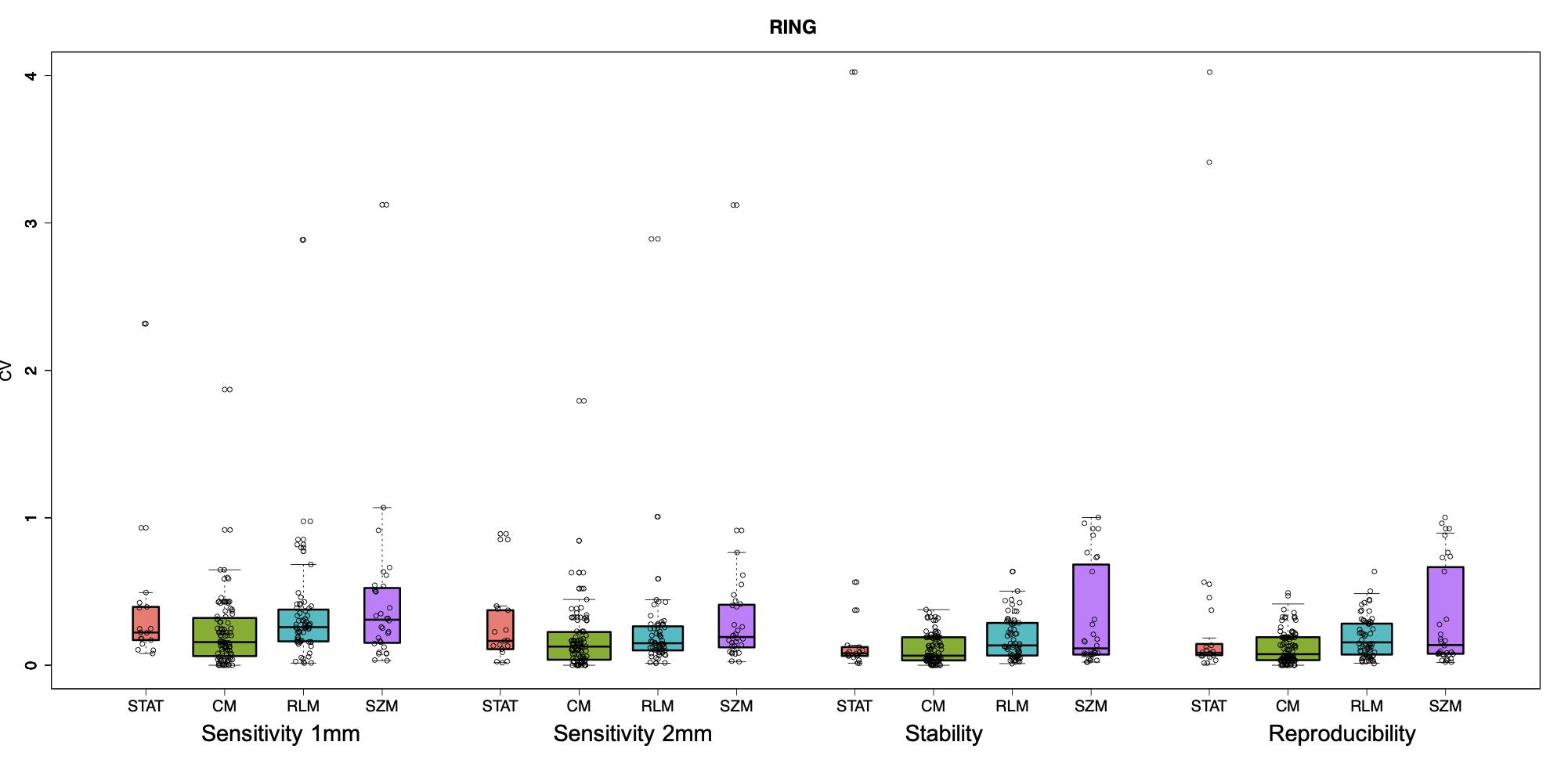

Supplement: Supplementary file 1 [file cancers-13-03835-s001.zip › Figure S4.jpg]

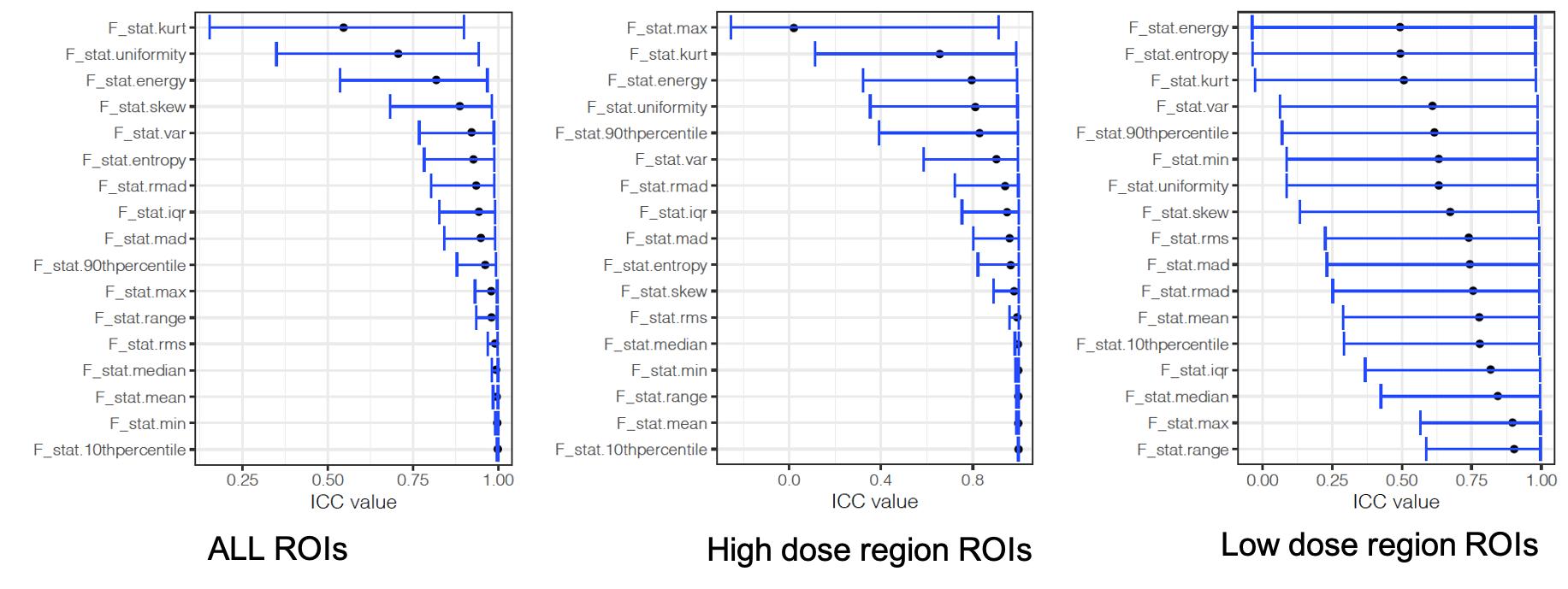

Supplement: Supplementary file 1 [file cancers-13-03835-s001.zip › Figure S5.jpg]
